# Experimental verification of principal losses in a regulatory particulate matter emissions sampling system for aircraft turbine engines

**DOI:** 10.1080/02786826.2021.1971152

**Published:** 2021-11-08

**Authors:** D. B. Kittelson, J. Swanson, M. Aldridge, R. A. Giannelli, J. S. Kinsey, J. A. Stevens, D. S. Liscinsky, D. Hagen, C. Leggett, K. Stephens, B. Hoffman, R. Howard, R. W. Frazee, W. Silvis, T. McArthur, P. Lobo, S. Achterberg, M. Trueblood, K. Thomson, L. Wolff, K. Cerully, T. Onasch, R. Miake-Lye, A. Freedman, W. Bachalo, G. Payne

**Affiliations:** a Department of Mechanical Engineering, University of Minnesota, Minneapolis, Minnesota, USA; b National Vehicle and Fuels Emissions Laboratory, Office of Transportation and Air Quality, U. S. Environmental Protection Agency, Ann Arbor, Michigan, USA; c Office of Research and Development, U. S. Environmental Protection Agency, Research Triangle Park, North Carolina, USA; d Formerly United Technologies Research Center, East Hartford, Connecticut, USA (retired); e Center for Excellence for Aerospace Particulate Emissions Reduction Research, Missouri University of Science and Technology, Rolla, Missouri, USA; f Aerospace Testing Alliance, Arnold Engineering Development Complex, Arnold Air Force Base, Tennessee, USA; g AVL-North America, Plymouth, Michigan, USA; h ARCADIS-U.S., Durham, North Carolina, USA; i National Research Council-Canada, Ottawa, Canada; j Boston College, Chestnut Hill, Massachusetts, USA; k TSI, Inc, Shoreview, Minnesota, USA; l Aerodyne Research, Inc, Billerica, Massachusetts, USA; m Artium Technologies, Sunnyvale, California, USA

## Abstract

A sampling system for measuring emissions of nonvolatile particulate matter (nvPM) from aircraft gas turbine engines has been developed to replace the use of smoke number and is used for international regulatory purposes. This sampling system can be up to 35 m in length. The sampling system length in addition to the volatile particle remover (VPR) and other sampling system components lead to substantial particle losses, which are a function of the particle size distribution, ranging from 50 to 90% for particle number concentrations and 10-50% for particle mass concentrations. The particle size distribution is dependent on engine technology, operating point, and fuel composition. Any nvPM emissions measurement bias caused by the sampling system will lead to unrepresentative emissions measurements which limit the method as a universal metric. Hence, a method to estimate size dependent sampling system losses using the system parameters and the measured mass and number concentrations was also developed (SAE 2017; SAE 2019). An assessment of the particle losses in two principal components used in ARP6481 (SAE 2019) was conducted during the VAriable Response In Aircraft nvPM Testing (VARIAnT) 2 campaign. Measurements were made on the 25-meter sample line portion of the system using multiple, well characterized particle sizing instruments to obtain the penetration efficiencies. An agreement of ± 15% was obtained between the measured and the ARP6481 method penetrations for the 25-meter sample line portion of the system. Measurements of VPR penetration efficiency were also made to verify its performance for aviation nvPM number. The research also demonstrated the difficulty of making system loss measurements and substantiates the E-31 decision to predict rather than measure system losses.

## Introduction

Environmental and global climate concerns about the fine particulate matter (PM2.5) emissions from commercial aircraft engines come from well-known health effects related to exposure to PM mass (U.S. EPA - United States Environmental Protection Agency 2019; Kärcher 2016; Burkhardt, Bock, and Bier 2018; Masiol and Harrison 2014; Jonsdottir et al. 2019; Yim et al. 2015). Because of the expected increased use of air travel over the next 20 years (United States Department of Transportation and Bureau of Transportation Statistics 2021; Masiol and Harrison 2014) there has been a concerted effort by the International Civil Aviation Organization (ICAO) to regulate PM emissions from aircraft gas turbines engines with measures more directly related to adverse health effects than the previously used Smoke Number standard. In 2020 the ICAO adopted amendments to its Annex 16, Volume II and the Environmental Technical Manual (ETM) to include a nonvolatile PM (nvPM) emissions standard for commercial aircraft gas turbines engines with a thrust greater than 26.7kN (ICAO 2017). These documents also specify requirements for the sampling and measurement for nvPM mass and number emissions which were based on SAE International Aerospace Recommended Practice (ARP) 6320 (SAE International 2018) and ARP6481 (SAE International 2019) as developed by the E-31 Aircraft Engine Gas and Particulate Emissions Measurement Committee. ARP6320 provides the nvPM number and mass sampling and measurement system specifications. In AIR5892B (SAE International 2012) the E31 standards committee defines nvPM as those particles present at the aircraft engine exit plane which do not volatilize when heated to 350 °C which omits the semi-volatile sulfur and organic particles formed in the downstream plume. The mass instruments measure black carbon which is a surrogate for nvPM mass that is used in ARP6320.

Aircraft gas turbines emit fine particulate matter consisting mainly of black carbon soot and organic carbon (OC), sulfates, and ash, with most of the mass found as carbon (Buseck et al. 2012; Schwartz et al. 2012). Large fractions of the OC and sulfates are not present as particles at the engine exhaust exit plane but form by gas to particle conversion as the exhaust dilutes and cools in the atmosphere (Petzold and Schröder 1998; Herndon et al. 2008; Onasch et al. 2009; Timko, Herndon, et al. 2010; Timko, Onasch, et al. 2010; Kinsey et al. 2011; Timko et al. 2013). Solid particles formed by gas turbine engines and present at the engine exit plane are very small, ranging from less than 10 to a few hundred nm in diameter (Durdina et al. 2014; Boies et al. 2015; Lobo, Durdina, et al. 2015; Lobo, Hagen, et al. 2015; Delhaye et al. 2017).

Because typical particle size distributions of particles generated by commercial aircraft engines have geometric mean diameters below 100 nm (and geometric standard deviations near 1.8) and the method (described below) specifies a long (≈ 35 m) sampling system containing sample dilution, volatile particle removal, and other transport equipment, the sampling system is prone to particle number concentration losses ranging from 50% to 90% (SAE International 2017; SAE International 2019; Crayford et al. 2011; Durdina et al. 2014; Brem et al. 2015; Durand, Crayford, and Johnson 2020). Long sampling lines are necessary because of harsh sampling conditions at the engine exit plane (e.g., velocities up to Mach 1, exhaust temperatures up to 900 °C). The major loss mechanisms are thermophoresis as the particles cool from exhaust gas temperatures to the front end sample line temperatures of 160 °C, diffusion and impaction to the walls in the long particle lines and other system elements such as the diluter and splitters, and thermophoretic and diffusional losses in the volatile particle remover (VPR). Coagulation can also occur prior to the sample being diluted if particle number concentrations are large enough (see Table 1). Although not a particle loss mechanism, the Condensation Particle Counter (CPC) particle counting efficiency must also be considered. ARP6481 specifies a method to estimate the particle losses in the ARP6320 sampling and measurement system using the system design and operation parameters and the nvPM mass and number concentration measurements in lieu of an actual particle size measurement.

To investigate both nvPM measurement variability and particle losses in ARP6320 compliant sampling systems, the U. S. Environmental Protection Agency (EPA), Office of Transportation and Air Quality (NVFEL) in Ann Arbor, MI in collaboration with EPA’s Office of Research and Development (ORD) in Research Triangle Park, NC and the U. S. Air Force’s Arnold Engineering Development Complex (AEDC) at Arnold Air Force Base, TN initiated the VAriable Response In Aircraft nvPM Testing (VARIAnT) research program (Kinsey et al. 2021). A part of the second VARIAnT test campaign (VARIAnT 2) was designed to evaluate the ARP6481 particle penetration calculation methods through direct measurement of particle penetrations through the 25-meter line portion of the system. VPR penetrations were also determined and compared with manufacturer’s specifications. In contrast to other particle penetration measurements (Durdina et al. 2017) of the ARP6320 sampling system components, these penetration measurements were conducted in situ with gas turbine engine particulates and with the ARP6320 sampling system fully operational. This article describes the results of the sampling system particle loss research conducted during the VARIAnT 2 test campaign using a J85-GE-5 turbojet engine burning multiple fuels at the University of TN Space Institute’s Propulsion Research Facility at Arnold Air Force Base, TN.

## ARP6320 sampling system and loss mechanisms

**Figure 1.**
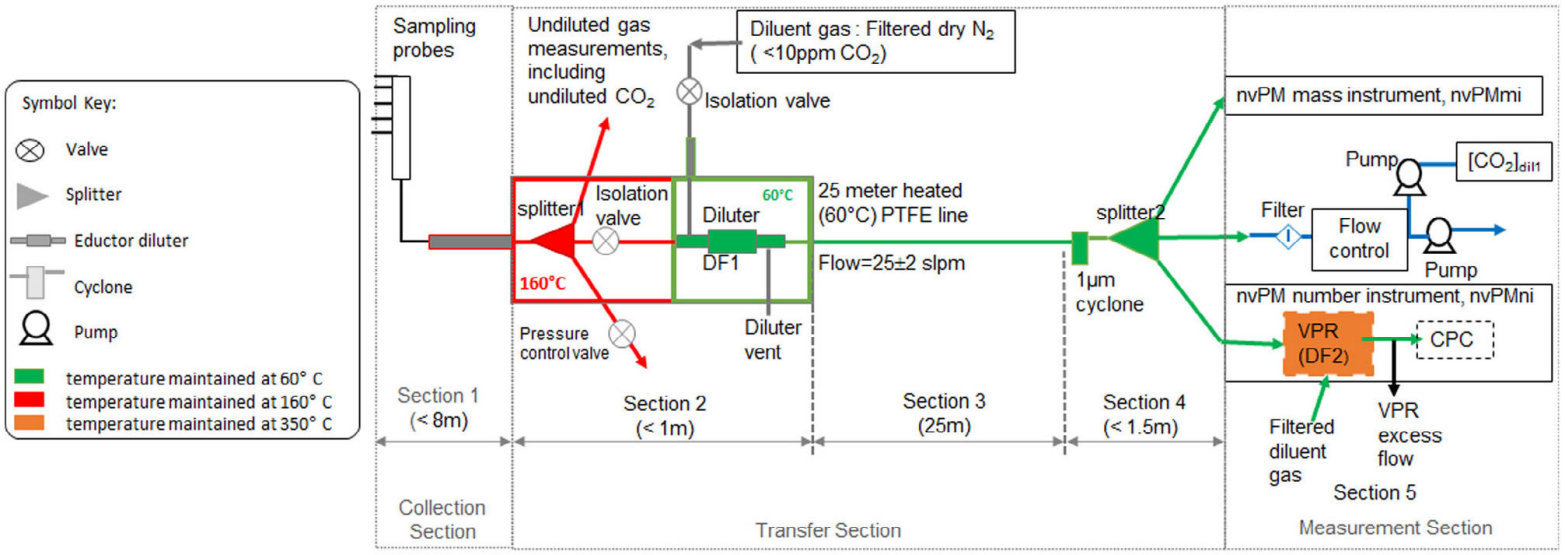
Diagram of ARP6320 sampling system. Standard conditions are 0 °C and 101.325 Pa.

Figure 1 is a schematic of the ARP6320 sampling and measurement system used in the current study. The following describes the sampling system, where particle losses occur within the system, and the approximate magnitude of particle loss for each sampling system segment.

Table 1 lists the sections of the sampling system and the loss mechanisms, i.e., thermophoretic, diffusional, and impaction (e.g., due to bends), that can occur for each section. The first part of the sampling system, Section 1 in Figure 1, is a sampling probe which is not temperature controlled located at the exit plane of the engine. From the probe, the sample flows through a heated (160 °C) sample line (up to 8 meters in length including the probe) to a three-way splitter (splitter1) also heated to 160 °C. In Section 2 of the sampling system, the splitter supplies sample for gaseous (total hydrocarbons, NO_x_, CO, CO_2_) emissions measurements, nvPM emissions measurement, and a third line to remove excess flow through the pressure control valve during periods of high thrust. From the splitter1 outlet the nvPM sample flows to an ejector diluter located within one meter of the splitter1 inlet which is also heated to 160 °C. During dilution, there is a sample temperature change from 160 °C to 60 °C which is mainly executed through the temperature controlled (60 °C) dilutor and N_2_ dilution gas. From the diluter outlet the nvPM sample flows at 25 ± 2 liters per minute (lpm) through the temperature controlled (60 °C) 25-meter line in Section 3 to a second splitter which provides sample to the number instrument, the mass instrument, and a third line for excess flow and the measurement of CO_2_. The sampling system components from the diluter outlet to the mass and number instrument inlets are all heated to 60 °C. The number instrument consists of a few short sample lines, a diluter, a volatile particle remover, and a condensation particle counter (CPC).

Penetration function calculations in this work are from the United Technologies Research Center (UTRC) particle transport model which has been described by Yook and Pui (2005), Wey and Liu (2008) and Liscinsky and Hollick (2010). The model was based on fundamental particle transport loss equations (e.g., Crane and Evans 1977; Friedlander and Johnstone 1957; Hinds 1999; Kim et al. 2005; Pui, Romay-Novas, and Liu 1987; Tsai et al. 2004; Tsai, Pui, and Liu 1990; Tsai and Pui 1990). Simplified calculations of thermophoretic losses were done using a model described by Kittelson and Johnson (1991) and discussed in further detail in the online supplementary information (SI).

Examples of calculated particle losses (due to diffusion, bends and thermophoresis) for each sampling system section are given in Table 2. These percent loss calculations used two lognormal particle size distributions with geometric mean diameters of 10 and 40 nm and both having a geometric standard deviation of 1.8. The sizes were selected to represent the range of what might be expected from modern aircraft turbine engines. The losses are based on the line dimensions, temperatures, and flows in the AEDC sampling system which is based on ARP6320 specifications and used in these experiments. In Table 2, undiluted refers to the line sections from the sampling probe tip to the inlet of the diluter. There can be significant thermophoretic losses when exhaust temperatures are above 160 °C in this section as the sample stream cools from turbine exhaust temperature to the 160 °C line temperature. For the J-85 engine used in our tests the exhaust temperature ranged from about 440 °C to 680 °C with corresponding thermophoretic losses ranging from 17 to 26%. These losses are essentially size independent for this range of particle diameters (D_p_ < 100 nm) and can be calculated from the inlet and outlet carrier gas absolute temperatures, T_inlet_ and T_outlet_, (Kittelson and Johnson 1991 and see also SI) as follows:

(1) $$\eta ={\left(\frac{T_{outlet}}{T_{inlet}}\right)}^{0.38}$$

Equation (1) is a simplified model based on well mixed plug flow through a pipe. On the other hand, as may be seen from Table 2, losses in the rest of the sampling system are size dependent. Additionally, as can be seen from Table 2, the majority of the particle losses occur in the 25-meter line and the VPR.

In ARP6841, the VPR penetrations are calculated from a fit to measured penetrations at four particle diameters, as specified in ARP6320. The fit includes diffusional losses (e.g., Hinds 1999; Yook and Pui 2005) and constant, size independent thermophoretic losses of Equation (1). For fully developed laminar flow through a circular tube, the particle penetration due to diffusional particle losses, *η*(*diffusion*) depends on the dimensionless deposition parameter (Hinds 1999),

(2) μ=DL∕Q

where *D* is the size and temperature dependent particle diffusion coefficient, *L* is the effective tube length, and *Q* is the actual volumetric flow rate. Thus, the penetration through the VPR is,

(3) η=η(diffusion)×η(thermophoresis)

The only unknowns in this expression are the effective length and the constant thermophoretic loss term.

In the 25-meter heated sampling line connecting the diluter to the measurement section, particle losses have been estimated using the UTRC calculator and are mainly due to diffusion. Losses associated with bends, inertia, and electrostatic interactions make up less than 2% of the losses over the size range measured. Except for the probe to diluter section of the sampling system, thermophoretic losses were negligible because the sampling line temperature was controlled to 60 °C.

The focus of this article is an evaluation of the losses in the 25-meter line. They were determined experimentally and compared with the calculation methods in ARP6481. The losses in the VPR were also evaluated and compared to those predicted by the expression described above which is used in ARP6481. However, particle concentrations downstream of the VPR are quite low leading to high uncertainty. Thus, these measurements were only done as a consistency check. This is discussed in detail in the results.

## Experimental

**Figure 2.**
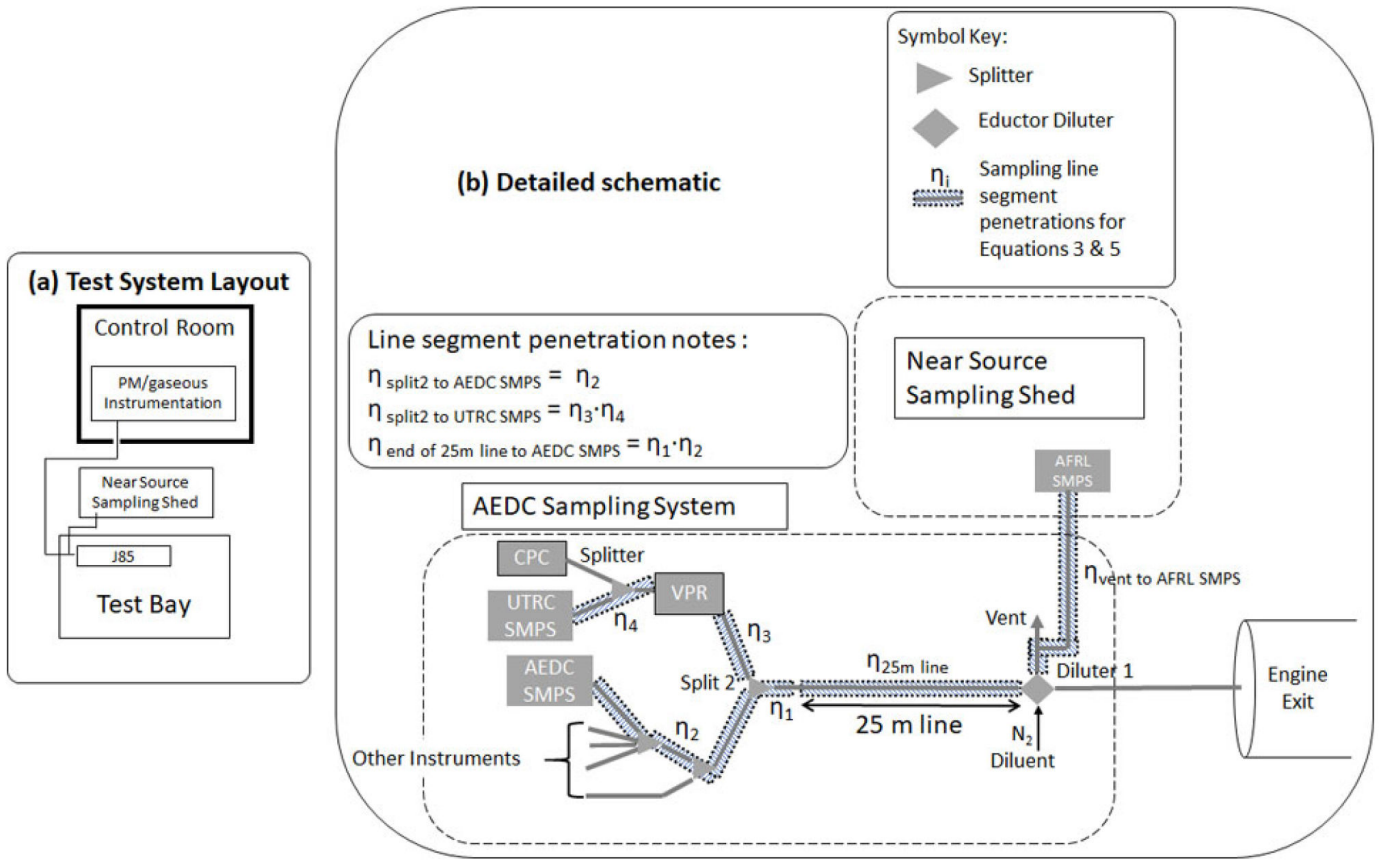
Schematics of (a) the AEDC sampling system, near source sampling shed, and the J85 engine test bay at the University of Tennessee Space Institute’s Propulsion Research Facility and (b) more detail with the locations of the size distribution measurements shown.

Sampling system losses were determined from particle size distributions measured at specific locations in the sampling system as shown in Figure 2. The size distribution measurements were made with TSI Scanning Mobility Particle Sizers (SMPSs) placed at the diluter vent, just downstream of the end of the 25-meter line, and in the VPR excess flow. Prior to the start of the test campaign the SMPSs underwent a comprehensive evaluation as described in the SI. In addition, daily comparisons of the SMPSs were conducted using a portable dioctyl sebacate (DOS) aerosol source (Leong et al. 1982; Liu and Lee 1975) and a TSI NanoScan SMPS, when available, was used to monitor relative concentration as the system was moved from instrument to instrument. The sampling system 25-meter carbon-impregnated Teflon line (with diameter of 0.80 cm) and VPR size dependent penetrations functions were determined from the particle size distributions (normalized to one another as explained below) measured upstream and downstream of the 25-meter line and the VPR, respectively. These were then compared to the penetration functions determined from the calculation methods used in the UTRC model and SAE ARP6481 using measured sampling system line dimensions, bend angles, temperatures, and flows and the fitted VPR penetration curve.

Specific components of the sampling system studied here for transport losses are the 25-meter heated line connecting Diluter 1 to the measurement section of the sampling system and the volatile particle remover (VPR) in the number measurement leg of the sampling system. The VPR consists of a diluter followed by a catalytic stripper (e.g., Abdul-Khalek and Kittelson 1995; Swanson and Kittelson 2010). The catalytic stripper contains a heated (typically to 350 °C) flow-through ceramic monolith consisting of many parallel channels that are coated with platinum, palladium, and/or rhodium. Particle diffusion losses in the channels and the thermophoretic loss in the downstream cooling section are predictable from the catalytic stripper design and operational parameters (Swanson et al. 2013). Combining all the sampling system penetrations, multi-plicatively, to determine a total sampling system loss penetration, allows the particle size distributions to be corrected to their upstream values.

### Equipment layout and instrumentation

A commercially available ARP6320-compliant sampling system, manufactured by AVL in Graz, Austria (AVL, 2021) and owned by AEDC was used to sample the exhaust from a J85-GE-5 (J85) turbojet engine. The sampling system was connected to a non-heated probe located at the center-line of the J85 exhaust nozzle (Figure S1 of the SI). A separate near-source sampling system housed in a portable enclosure located adjacent to the test bay (Figure 2) housed the AFRL SMPS to measure the nvPM size distribution upstream of the 25-meter heated line. This line originated from the vent outlet of the Dekati diluter (see Figure 2). The AEDC SMPS was connected to a splitter at the end of the 25-meter sampling line as shown in Figure 2. Finally, the UTRC SMPS was connected to the VPR excess flow to find the VPR penetrations for comparison with fitted penetration curve based on manufacture’s data. All SMPSs used during the VARIAnT 2 test campaign are listed in Table 3.

TSI Aerosol Instrument Analyzer (AIM®) Version 10.2 software was used to determine the size distributions (see section 2.1 of the SI). Each SMPS recorded a scan every 3 min. Two 500 μCi ^210^Po neutralizer strips (NRD, LLC, Grand Island, NY, USA) were used as the bipolar charger in each of the SMPSs with either the 3080 and 3082 differential mobility analyzers (DMAs). The portable NanoScan used a unipolar diffusion charger, radial DMA and compact CPC.

### Evaluation of SMPSs

The SMPS quality assurance tests are described below with further details described in the SI. Prior to the VARIAnT 2 test campaign, two of the SMPSs (i.e., the NRMRL and NVFEL SMPSs) were sent to TSI for servicing and then to the University of Minnesota for pretest evaluations. In addition, the AFRL, UTRC and AEDC SMPSs were also sent to the University of Minnesota for pretest evaluations. In the pretest evaluation at the University of Minnesota, CPC flow, classifier flow, CPC analog voltage output (voltage to the DMA column), and zero checks were performed on each instrument. Additionally, instrument inter-comparisons of particle number concentration and size distribution parameters were performed with a variety of aerosol source types (i.e., John Deere 4045 diesel engine exhaust, silver particles generated from an oven, and DOS and polystyrene latex sphere aerosols generated with an atomizer).

Size distribution parameters measured by the different SMPSs were also compared. The diesel engine source was operated at 3 conditions, 2 tests at each, giving particles in the 30 to 60 nm size range. The SMPS measured total particle number concentrations with agreement of better than ±10% and measured number mode diameters within ±2 nm for each condition. The DOS source was adjusted to give nominally 50 nm particles and tested twice. The SMPSs showed agreement of better than ± 20% for particle number concentration and measured geometric mean diameters within 2 nm. The silver aerosol source was used at 2 nominal sizes, 8 and 15 nm with 2 tests at each size. The SMPSs showed agreement of particle number concentrations within 30% and geometric mean dimeters agreement within ±2 nm. PSL measurements showed sizing agreements within ±4 nm of the nominal 200 nm PSL for all SMPSs, except the NRMRL SMPS which agreed within 5% of the expected peak diameter.

At AEDC, SMPS inter-comparison tests were done for quality control purposes both during the testing and at the end of the test campaign. Prior to the start of each day’s testing, co-located SMPS measurements of a DOS aerosol size distribution generated by a constant output atomizer (Leong et al. 1982; Liu and Lee 1975) were made which sometimes included the TSI NanoScan® SMPS. These tests compared the DOS aerosol size distributions measured by 2 SMPSs in a given sampling system along with the NanoScan® SMPS (e.g., Figure S6 in the SI). The NanoScan® SMPS was portable, could be used in each sampling system, and could thus be used as a consistency check between all SMPSs. These tests gave daily SMPS comparisons of the particle number concentrations, geometric mean diameters, and geometric standard deviations for the two SMPSs (sometimes with the TSI NanoScan®) at each of the three SMPS sampling locations (e.g., Table S3 in the SI). In the daily size checks, the instruments showed very good day to day repeatability with concentrations usually within about 5% and size within about 2 nm.

Finally, a post-test comparison was made of all the SMPSs simultaneously sampling a nominally 60 nm DOS aerosol with 3 repeats. Total number concentrations measurements agreed within ±20%. Five of the 6 SMPSs measured geometric mean diameters within ±2 nm, but one read consistently 3 to 5 nm low.

### SMPS measurements

As shown in Figure 2, for the 25-meter sample line and the VPR, size distributions were measured simultaneously with two SMPSs, one upstream and one downstream of the sampling system component during steady-state operation, i.e., at a constant Power Level Angle (PLA) value, of the J85. The operating points of the J85 were varied by fuel type and power lever angle (thrust). The size distributions used in the calculations are the average of multiple 3-min SMPS scans over the course of a steady-state operation point.

The upstream and downstream SMPS sampling locations could not be placed directly at the beginning and end of each of these sampling system components due to the constraints of the ARP sampling system. To make the SMPS measurements feasible additional line lengths had to be added. The theoretical penetrations for these additional line lengths were also estimated using the UTRC tool which were then used to normalize the upstream and downstream size distributions for these additional particle losses. As two different instruments were used to make simultaneous upstream and downstream measurements, considerations for differences in size dependent instrument responses were taken into account. In particular, instrument normalizations or instrument response ratios for each SMPS size bin were determined using the DOS aerosol instrument comparisons. Details on how the size dependent instrument response ratio was determined are in the SI.

### 25-Meter line penetrations

The 25-meter line size dependent penetration was calculated from the measured upstream size distribution, f_AEDC_(D_p_), and downstream size distribution, f_AFRL_(D_p_), the ratio of additional line losses to each SMPS, and the ratio of instrument responses for the two SMPSs as shown in Equation (5). The ratio of additional particle losses and the ratio of instrument responses were both near unity for particle diameters larger than 10 nm as shown in the SI.

(4) $$\eta_{25m\;line}(D_{p})=\left(\begin{array}{c} ratio\;of\;measured\\ size\;distributions \end{array}\right)\;\times \left(\begin{array}{c} ratio\;of\;additional\\ particle\;losses \end{array}\right)\times \left(\begin{array}{c} ratio\;of\;instrument\\ responses \end{array}\right)$$

(5) η25mline(Dp)=fAEDC(Dp)fAFRL(Dp)×[∕ηendof25mlinetoAEDCSMPS(Dp)1][∕ηventtoAFRLSMPS(Dp)1]×NAFRL∕AEDC(Dp)

where f_AEDC_(D_p_) = the size distribution (dN/dLogD_p_) measured with the AEDC SMPS downstream of the 25-meter sample line, f_AFRL_(D_p_) = the size distribution (dN/dLogD_p_) measured with the AFRL SMPS upstream of the 25-meter sample line, *η*_end of 25 m line to AEDC SMPS_(D_p_) = the calculated penetration of the short sample line connecting the end of the 25-meter sample line (split 2) to the inlet of the AEDC SMPS (e.g., Figure 2b), *η*_vent to AFRL SMPS_(D_p_) = the calculated penetration of the short sample line connecting the beginning of the 25-meter sample line to the inlet of the AEDC SMPS (e.g., Figure 2b), N_AFRL/AEDC_(D_p_) = an SMPS to SMPS size dependent number concentration normalization ratio determined from the post-test equivalent DOS sample supplied to each SMPS to account for differences in different SMPS instrument responses (see SI).

### VPR penetrations

The VPR size dependent penetration was calculated as shown by Equation (7) from the upstream AEDC SMPS size distribution, f_AEDC_(D_p_), downstream UTRC SMPS measured size distributions, f_UTRC_(D_p_), the ratio of additional line losses to each SMPS, the ratio of instrument response for the two SMPSs and correction for dilution. The ratio of additional particle losses and the ratio of instrument responses were near unity for particle diameters larger than 10 nm as shown in the SI.

(6) $$\eta_{\text{VPR}}(D_{p})=\left(\begin{array}{c} ratio\;of\;measured\\ size\;distributions \end{array}\right)\;\;\times \left(\begin{array}{c} ratio\;of\;additional\\ particle\;losses \end{array}\right)\;\;\times \left(\begin{array}{c} ratio\;of\;instrument\\ responses \end{array}\right)\;\;\times \left(\begin{array}{c} dilution\\ correction \end{array}\right)$$

(7) ηVPR(Dp)=fUTRC(Dp)fAEDC(Dp)×[∕ηsplittertoUTRCSMPS(Dp)1][∕ηsplit2toAEDCSMPS(Dp)1]×NAEDC∕UTRC(Dp)×DF2

where f_AEDC_(D_p_) = the size distribution (dN/dLogD_p_) measured with the AEDC SMPS upstream of the VPR, f_UTRC_(D_p_) = the size distribution (dN/dLogD_p_) measured with the UTRC SMPS downstream of VPR, *η*_split2 to AEDC SMPS_(D_p_) = the calculated penetration of the short sample line connecting the outlet of the split2 splitter to the inlet of the AEDC SMPS (e.g., Figure 2b), *η*_splitter to UTRC SMPS_(D_p_) = the calculated penetration of the short sample line connecting the outlet of the VPR to the inlet of the UTRC SMPS and the section from split2 to the VPR inlet (e.g., Figure 2b), N_AEDC/UTRC_(D_p_) = an SMPS to SMPS size dependent number concentration normalization ratio determined from the post-test equivalent DOS sample supplied to each SMPS (see section the SI), DF2 = Number instrument dilution factor which reduces the number concentrations measured by the CPC by about a factor of 900 to allow the CPC to stay in single particle count mode, avoiding the CPC coincidence correction

## Results and discussion

### 25-Meter line penetration results

**Figure 3.**
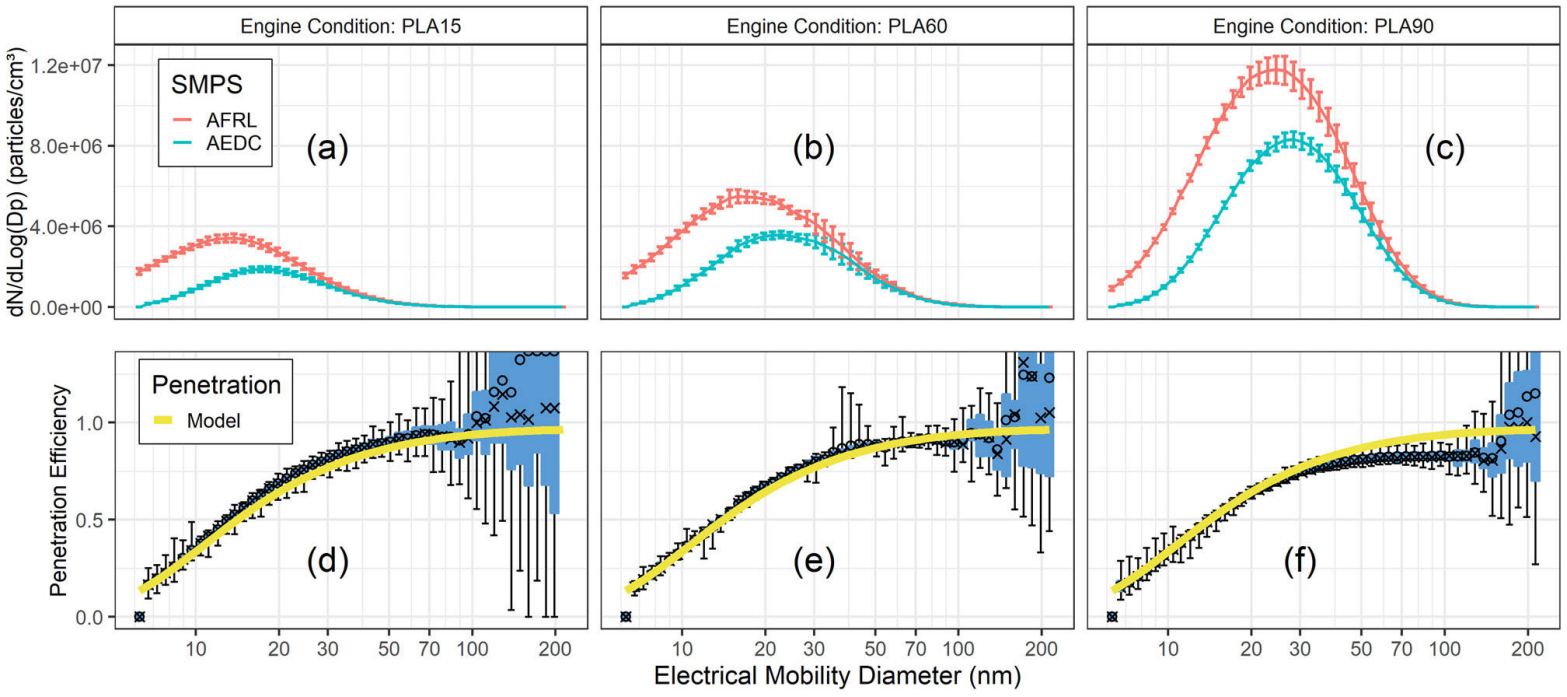
Measured SMPS particle size distributions from the J85 engine at different PLAs with Jet-A fuel are shown in (a) to (c) with the penetration efficiencies computed from the measured size distributions compared to theoretical penetrations provided in (d) to (f). The distributions represent the average of 96, 14, and 66 scans for PLA15, PLA60, and PLA90, respectively.

Overall, there were 19 different J85 test conditions over 5 days that varied in fuel and thrust. Five of the 19 test conditions showed unusual shifts in the penetration curves, all of which occurred on the same day. The remaining 14 test conditions showed consistent penetration curve results and 3 representative examples are shown in Figure 3. The size dependent penetration efficiency through the 25-meter line was computed from Equation (5) using the measured size distributions from the AEDC and AFRL SMPSs. Figure 3 shows the measured size distributions and resulting penetration curves computed from Equation (5) for engine thrust which is determined by PLAs of 15, 60, and 90 degrees while operating on Jet-A fuel. The size distributions shown in Figures 3a to c are the averages with standard deviations of multiple scans over the test condition. The penetrations computed from Equation (5) are shown in Figures 3d to f and are compared to the modeled values for penetration from the UTRC line loss model. A box and whisker plot is shown for each particle diameter. The whiskers at each particle diameter show the computed penetration minimum and maximum value. Additionally, the computed penetrations for the 1st and 3rd quantile are represented by the blue box. The open circles represent the mean and the x’s show the median computed penetration values.

The measured size distributions (Figures 3a, b, and c) show that both particle number concentrations and particle size increase with increasing engine power. The measured penetrations shown in Figures 3d, e, and f are more variable for larger particle diameters where the measured size distributions in both upstream and downstream SMPSs had fewer counts as illustrated in the top panel. The variability in the computed penetration is shown by the larger spread of the box and whisker plots and the mean and median values deviating from one another especially at particle diameters >100nm. Overall, the measured median penetrations between 7 and 120 nm for the 25-m line agree well with values predicted by the UTRC line loss model to within ±15%. However, there is a slight decrease in the measured particle penetrations relative to calculations associated with higher thrust and larger particles that we do not understand. We considered thermophoresis, electrostatic losses, ejector diluter vent flow, poor mixing in the exit of the ejector diluter and none of the explanations was consistent with the observed results.

### VPR penetration results

The size dependent penetration efficiency through the VPR was computed from Equation (7) using the measured size distributions from the UTRC and AEDC SMPSs. The UTRC SMPS is located downstream of the number instrument VPR and diluter. The VPR system is designed to keep the concentration at the inlet to the CPC in the single count range (well below 10,000 particles/cm^3^). This leads to measured particle concentrations downstream of the VPR as low as about 1500 particles/cm^3^ resulting in variations in individual TSI Scanning Mobility Particle Sizers SMPS size bin concentrations of up to a factor of 2 or more.

**Figure 4.**
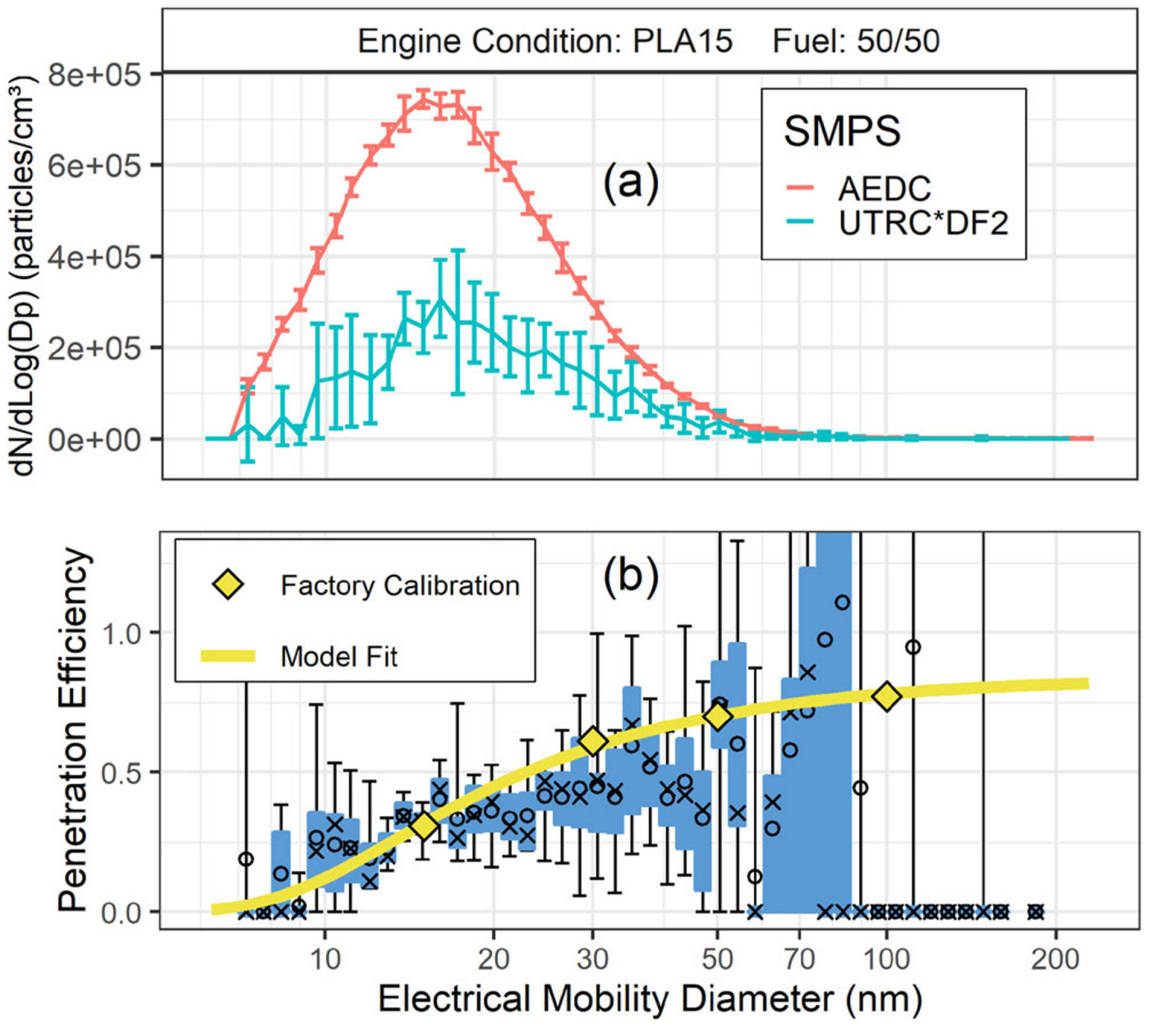
VPR penetration measurements, comparisons with manufacturer, and UTRC model. The top panel (a) shows the particle size distributions at PLA 15 when a 50/50 blend of Jet-A and Camelina fuels was used. The AEDC SMPS size distribution is plotted as measured and the UTRC SMPS is corrected for the dilution in the VPR. The line represents the average size distribution and the error bars show the standard deviation. The error bars highlight the variability. The bottom panel (b) shows the penetration efficiency obtained from the measured size distributions compared to the modeled penetration for the VPR. The distributions in (a) represent the average of 7 scans.

Figure 4 shows the upstream and secondary dilution corrected (DF2) downstream size distributions measured at PLA 15 using a 50/50 blend of Jet-A and Camelina fuels, resulting penetration data, and the modeled penetration curve based on manufacturer supplied VPR penetrations at 15 nm, 30 nm, 50 nm, and 100 nm. The size distributions shown in Figure 4a are the average and standard deviations of 7 scans over the test condition. The penetrations determined from the measured sized distributions and computed with Equation (7) are shown in Figure 4b. For each particle diameter a box and whisker plot shows the first and third quantiles and the error bars indicate the penetration minimum and maximum as described above. The open circles represent the mean and the x’s represent the median penetration values determined from the measurements. The solid, yellow line in Figure 4b represents the ARP6481 VPR model.

The VPR penetrations in Figure 4b calculated from the measured size distributions had much higher variability as compared to the 25-meter sample line results. As mentioned above, the downstream size distribution measured by the UTRC SMPS is extremely dilute (overall dilution ratio up to ~800) resulting in very low particle number concentrations (10^4^ particles/cm^3^ or less) downstream of the VPR. Several cases showed unusual penetration curves. These results may have been compromised by low concentrations and lab air entrainment into the sample due to the location and flow rate coming out of the VPR vent

VPR manufacturers test under controlled laboratory conditions typically using monodisperse test aerosols (lab burner soot) at the four individual particle diameters and at concentration levels much higher than the VARIAnT 2 in-situ measurements made. Although, the downstream particle concentration measured by the UTRC SMPS was highly dilute and variable, the general trends for VPR penetrations agreed with manufacturer calibration results. Our tests demonstrate the additional difficulty of making these in situ measurements as other investigators (e.g., Durdina et al. 2017) have made these measurements while not using the full ARP6320 sampling system, nor measuring nvPM emissions from a gas turbine engine.

## Summary and conclusions

In situ size dependent penetration efficiencies through the 25-meter line on the AEDC regulatory compliant nvPM sampling system was measured using a pair of well-characterized SMPS’s and while operating the ARP6320 sampling system to measure nvPM from a J85 gas turbine engine. The penetration efficiency agreed well with values predicted by the UTRC line loss model to within about ±15% for particle mobility diametersbetween 7 and 120 nm. Measured penetration efficiency curves shifted very slightly downward (i.e., Approximately a maximum of 15%) compared to the theory for the larger particle diameters (≳50nm) and higher engine thrusts. In situ particle loss measurements for the VPR were difficult to make due to low particle sample concentrations at the SMPS downstream of the VPR, but were found to be generally consistent with manufacturer’s specifications and the VPR model provided in ARP6481 (SAE International 2019).

Additionally, particle size and number instrument diagnostic test protocols for the SMPSs (differential mobility analyzers and CPCs) were established. Most of the SMPSs in the study exhibited consistent, comparable, and reproducible behavior throughout. This was able to be verified with the pretest, daily, and post test quality control checks performed. These checks are very much needed to ensure the collection of high quality, reproducible size measurements which should be conducted in any future similar tests. Also, without multi-day, replicate testing it would have been impossible to identify anomalous results.

Despite careful planning and execution of these experiments, a few inconsistent results demonstrate the difficulty of accurate in-use penetration measurements. Calculating the line losses with the sampling system parameters rather than measuring the losses for each gas turbine engine test campaign is a more robust approach.

Potential future studies could include examining aspects of the system that are not included in the ARP6481 tool, for example, characterizing losses in the diluter and losses in splitters, especially when the differences in the splitter leg flows are large. In addition, the loss tool may be used to predict front end losses, but these losses have not been experimentally verified.

## Supplementary Material

supplementary material

## Figures and Tables

**Table 1.** Loss mechanisms in each section of the ARP6320 sampling system.

Sampling System Section	Particle sampling line components	Temperature (°C)	Particle loss mechanisms			
			Coagulation	Impaction to walls	Diffusion to walls	Thermophoresis
Section 1	Unheated probe and probe tips, heated line	>900 to 160^a^	**X** ^b^	X	X	X
Section 2	Heated lines, junctions, and Splitter 1	160	**X** ^b^	X	X	X
	Heated diluter	160 to 60	N/A	X	X	X
Section 3	Heated 25-meter line	60	N/A	X	X	N/A
Section 4	Heated lines, junctions, 1 um cyclone, and Splitter 2	60	N/A	X	X	N/A
Section 5	nvPM number measurements VPR & CPC	60 to 350 to 60	N/A	X	X	X
	nvPM mass measurement	60	N/A	X	X	N/A

a May be less than 160°C for mixed flow engines.

b May reduce number concentrations in this section by 5% or more when particle concentrations at the engine exit plane are ≳5 × 10^7^ particles/cm^3^.

**Table 2.** Typical loss estimates for the AEDC sampling system as a percent of the initial number, N_inlet_, and mass, M_inlet_, of particles at the inlet of each sampling system section. N_exit_ and M_exit_, are the number and mass of particles, respectively, at the exit of the sampling system section. Refer also to Figure 1 and Table 1 to relate these losses to specific sampling system segments and particle loss mechanisms. Thermophoretic losses are not included in this table but are discussed in the text.

Engine exhaust plane geometric mean diameter, D_g_, and standard deviation, *σ*_g_	Number reductions (%), (1-N_exit_/N_inlet_) × 100					
	Sections 1 & 2 undiluted	Section 3 25 m line	Section 4 Line to number instrument	Section 5		total
				VPR	CPC	
D_g_=10nm, *σ*_g_=1.8	21	50	18	67	13	91
D_g_=40nm, *σ*_g_=1.8	8	21	7	41	1	61
Engine exhaust plane geometric mean diameter, D_g_, and standard deviation, *σ*_g_	Mass reductions (%), (1-M_exit_/M_inlet_) × 100					
	Sections 1 & 2 undiluted	Section 3 25 m line	Sections 4 & 5 Line to mass instrument		total	
D_g_=10nm, *σ*_g_=1.8	12	31	6		43	
D_g_=40nm, *σ*_g_=1.8	3	8	1		12	

**Table 3.** SMPS equipment list and location.

Location/ Sampling System	Instrument Owner	Instrument ID	Classifier Model	DMA Model	CPC Model	Software Version
AEDC	Air Force Research Laboratory	AFRL	3080	3081	3776 High Flow	AIM® 9^a^
	Arnold Engineering Development Complex	AEDC				
	United Technologies Research Center (Classifier & DMA) & University of Minnesota (CPC)	UTRC				
MST	EPA Office of Research and Development	NRMRL^b^				
	EPA National Vehicle and Fuel Emissions Laboratory	NVFEL				
Near Source^c^	TSI Incorporated	TSI	3082			AIM® 10.2
Various	TSI Incorporated	NanoScan	integrated	radial	Isopropanol based	NanoScan®

a All data from these instruments were post-processed through the AIM 10.2 software.

b Failed during campaign.

c See Kinsey et al. 2021.
