# Supplementary material for: Experimental verification of principal losses in a regulatory particulate matter emissions sampling system for aircraft turbine engines
Source: Aerosol Sci Technol. Author manuscript; Available in PMC 2022 May 19. (PMC9118390; doi:10.1080/02786826.2021.1971152)
Supplement: supplementary material [file NIHMS1802017-supplement-supplementary_material.docx]

**Supplemental Information**

for

**Experimental Verification of Particle Losses in a Regulatory PM Emissions Sampling System for Aircraft Turbine Engines**

# Sample probes and rake

During VARIAnT 2, a four-probe rake attached to a horizontal traversing table was used to collect the samples provided to the two ARP-compliant sampling systems, the near-source sampling system, and the gaseous pollutant monitoring bench. Photos of this equipment are shown in Figure S-1.


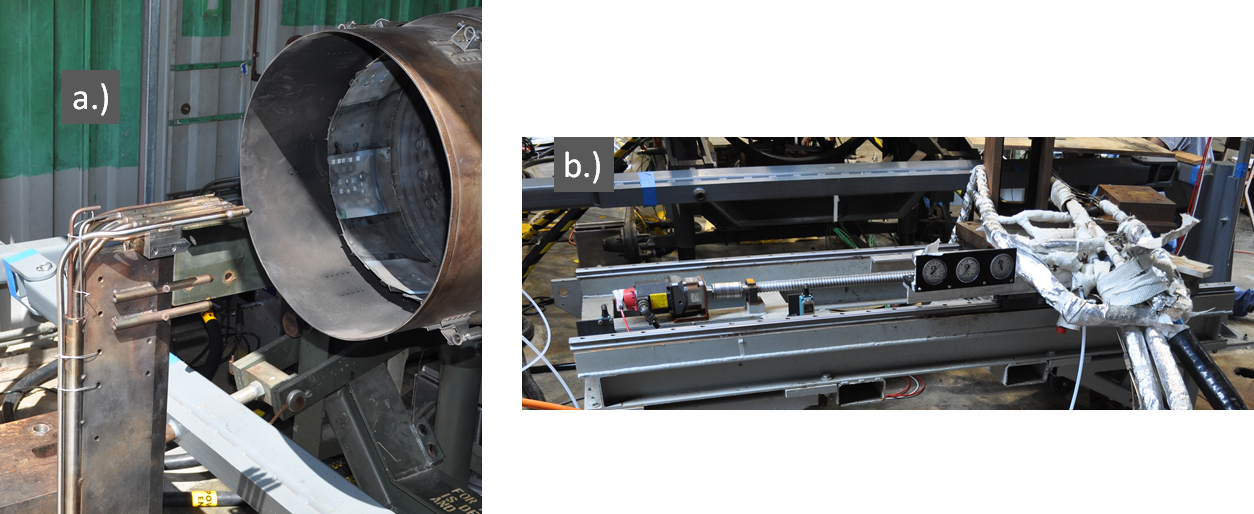


Figure S-1. Rake configuration for J-85 testing. a.) the four probes mounted horizontally side by side at the engine exit and b.) traversing table mounting whereby the probes can be remotely moved horizontally across the engine exit plane.

# SMPS Quality Control Procedures

## SMPS software

During the test campaign, there were multiple models of SMPSs with differing versions of operating software [in particular, TSI Aerosol Instrument Analyzer (AIM^®^) Version 9 and TSI AIM^®^ Version 10] used to determine the size distributions. It was necessary to use AIM^®^ 9 for data collection with the TSI SMPS 3080 classifier models while AIM^®^ 10 was used with the newer TSI SMPS 3082 classifier model. Discrepancies were found in the size distributions when the diffusion correction was applied to the measured, raw size distributions with TSI AIM^®^ 9. The manufacturer determined that the TSI AIM^®^ 9 software had an error in its diffusional loss calculation. All data collected with TSI AIM^®^ 9 analyzed in this work was reprocessed in TSI AIM^®^ 10 software to avoid this problem.

## SMPS evaluations, inter-comparisons, and normalizations

Results of the particle penetration measurements were used to compare with theoretical calculations of particle penetrations. Hence, a thorough investigation of the instrument responses was made prior to the campaign, during testing, and after the tests were completed.

### Pre-campaign SMPS evaluations

Prior to the VARIAnT 2 test campaign, two of the SMPSs (i.e., the NRMRL and NVFEL SMPSs) were sent to TSI for servicing and then to the University of Minnesota (UMN) for pre-test evaluations. In addition, the AFRL, UTRC and AEDC SMPSs were also sent to the University of Minnesota for pre-test evaluations. In the pre-test evaluation at the University of Minnesota, CPC flow, classifier flow, CPC analog voltage output (voltage to the DMA column), and zero checks were performed on each instrument. In addition, particle concentration and size distribution checks that included instrument inter-comparisons and 4 different types of aerosols were performed. Schematics of the sampling configurations for these tests are in Figure S-2 through Figure S-5.


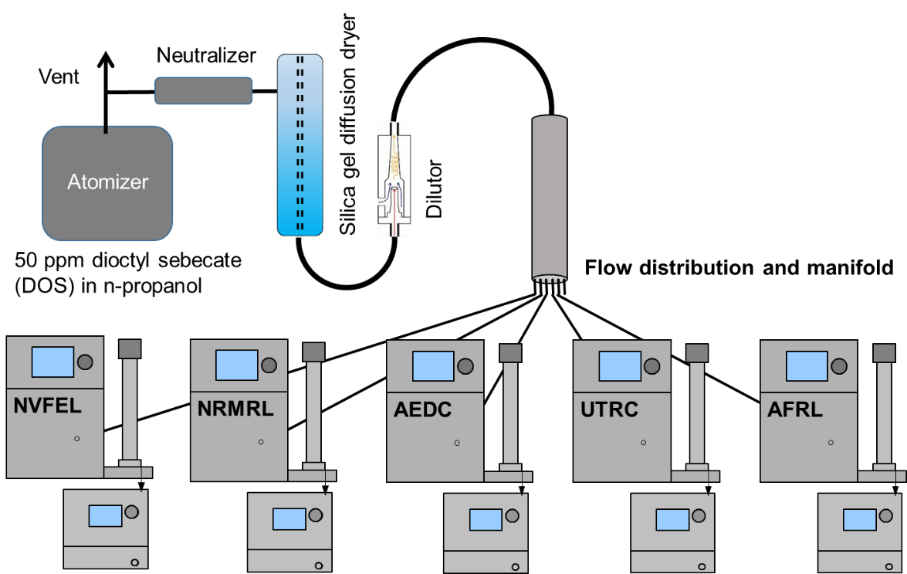


Figure S-2. SMPS DOS sampling configuration at the UMN.


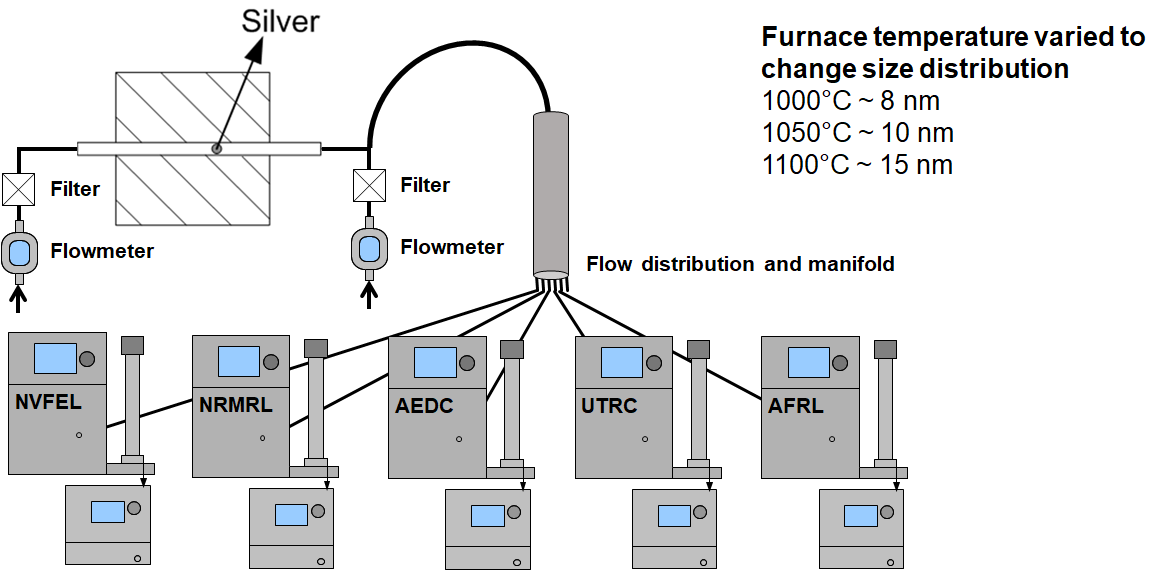


Figure S-3. SMPS silver aerosol sampling configuration at the UMN.


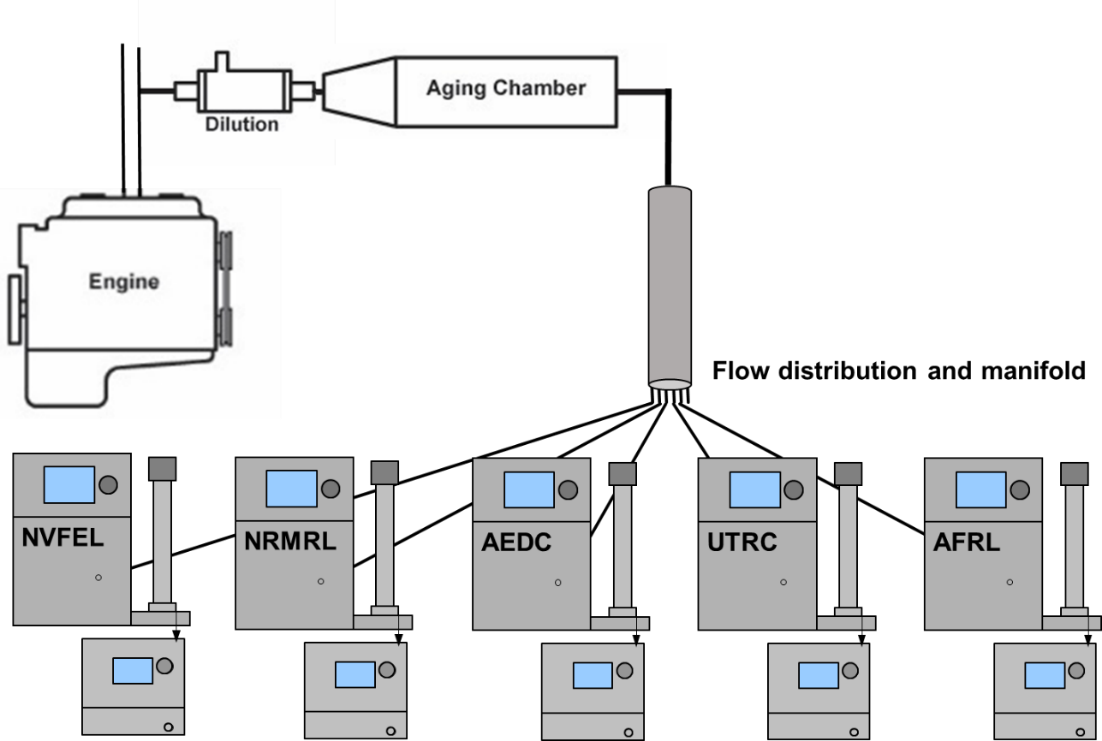


Figure S-4. Diesel soot sampling configuration for John Deere 4045 at the UMN.


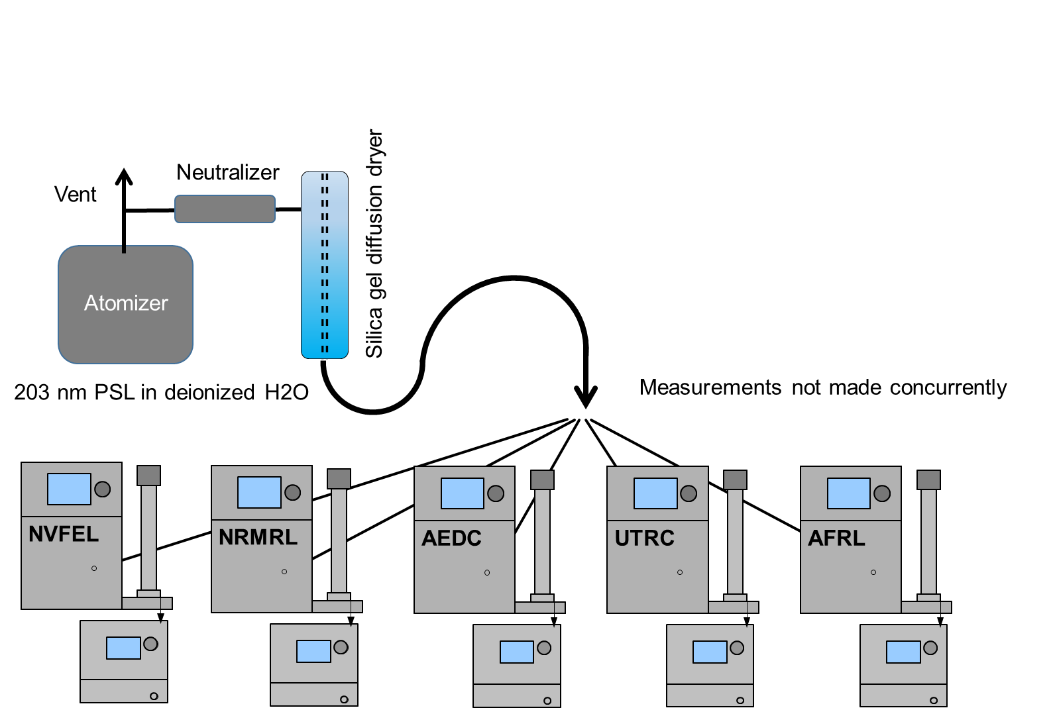


Figure S-5. Polystyrene Latex (PSL) sphere aerosol sampling configuration at the UMN.

The flow rates for the CPC, classifier inlet impactor, and DMA sheath air were measured for all 5 instruments and found to be within ±5% of the instrument specified flow rates (see Table S-1). Zero checks were performed for each instrument by connecting a HEPA filter at the inlet of the instrument and a measured concentration of less than 1 particle/cm³ averaged over 60 seconds was considered acceptable (see Table S-1). The neutralizer zero checks were performed to ensure there were no leaks in the ^210^Po neutralizer containers.

Table S-1: SMPS & CPC zero checks and flow readings (flow rates for TSI instruments are determined/calibrated at ambient temperature and pressure)

| Instrument ID | CPC zero | Instrument zero | Neutralizer zero | CPC flowrate  (lpm)^a^ | Impactor flowrate  (lpm)^b^ | Sheath flowrate  (lpm)^c^ |
| --- | --- | --- | --- | --- | --- | --- |
| **AEDC** | Passed | Passed | Passed | 1.50 | 1.43 | 14.8 |
| **NVFEL** | Passed | Passed | Passed | 1.51 | 1.45 | 14.4 |
| **NRMRL** | Passed | Passed | Passed | 1.52 | 1.46 | 15.0 |
| **UTRC** | Passed | Passed | Passed | 1.48 | 1.43 | 14.7 |
| **AFRL** | Passed | Passed | Passed | 1.50 | 1.43 | 14.9 |

^a^ Target flow rate for the CPC high flow setting is 1.50 lpm. Acceptable range is within 5% of the set point, 1.425 - 1.575 liters/minute.

^b^ Target flow rate for the Impactor flow rate for a CPC high flow setting is 1.50 lpm. Acceptable range is within 5% of the set point, 1.425 - 1.575 liters/minute.

^c^ Target flow rate for the Sheath flow was 15.0 lpm. Acceptable range is within 5% of the set point, 14.25 - 15.75 liters/minute.

Each SMPS was also evaluated with a polydisperse test aerosol consisting of a nebulized and dried solution of dioctyl sebacate (DOS) in isopropyl alcohol. The DOS solution concentration was varied to produce different size distributions, with approximately 50 ppm DOS in alcohol. The DOS aerosol particle concentrations were varied by dilution with compressed, filtered air. For the initial tests, each SMPS was connected to a sampling manifold as shown in Figure S-2. Two configurations with randomly configured sampling lines showed no apparent bias with the sampling system.

The DOS tests did reveal, however, that the UTRC SMPS was reading concentrations ~50% lower than the other SMPSs. This was traced to the CPC in the UTRC SMPS which was undercounting due to a blockage in the aerosol flow capillary (which is nominally 50 mL/min). This was addressed by removing and cleaning the capillary. The CPC was dissembled, cleaned, had its saturator wick replaced, and was then re-assembled. Subsequent comparison with DOS gave evidence that CPC issue was fixed because all SMPSs had concentrations within +/- 15% of each other.

A silver aerosol source (Figure S-3) was also used at the UMN to produce particles with geometric mean diameters of 8 nm, 10 nm, and 15 nm and allowed an instrument check with a lower range of particle diameters. The silver tests showed that the UTRC SMPS was not properly selecting smaller sized particles due to incorrect CPC analog voltage which sets the DMA voltage for specific particle diameters. The CPC analog voltage was measured for all instruments at 10 mV and 9.9 V with a NIST traceable voltmeter. Table S-2 shows the measured voltages for each CPC and that all CPCs except the NVFEL CPCs, had incorrect lower voltages. These incorrect CPC analog voltages were for DMA voltages that selected particles with diameters smaller than 20 nm. For the worst-case low‑end voltage error, instead of 10 mV, the UTRC CPC had an output voltage of 18.9 mV. This voltage difference would lead to a sizing error selecting particles nearly 8 nm larger. The analog voltage output was recalibrated for all CPCs except the NVFEL CPCs. Correcting the CPC analog output voltage was the single most important factor in achieving instrument sizing agreement among the five SMPSs.

Table S-2: CPC output voltage readings before calibration. The 10 mV and 9.9 V target voltages correspond to DMA particle diameter selections of approximately 6 and 225 nm, respectively, at the sheath 15 lpm, and sample 1.5 lpm flows used in these tests.

| Instrument ID | CPC analog output target low voltage setpoint = 10 mV^a^  (mV) | CPC analog output target high voltage setpoint = 9.90 V^b^  (V) |
| --- | --- | --- |
| **AFRL** | 9.05 | 9.90 |
| **NVFEL** | 9.88 | 9.90 |
| **NRMRL** | 8.85 | 9.90 |
| **UTRC** | 18.9 | 9.91 |
| **AEDC** | 9.80 | 9.90 |

^a^ Acceptable range is from 9.70 mV to 10.3 mV for the low voltage setpoint

^b^ Acceptable range is from 9.88 V to 9.91 V for the high voltage setpoint

After correcting for all instrument issues, particle size distribution comparisons at the UMN were performed for silver aerosols (repeats) and DOS and aerosol from a John Deere 4045 diesel engine as shown in Figure S-6, Figure S-7, and Figure S-8, respectively. The diesel soot was sampled in the configuration shown in Figure S-4 at 3 test conditions leading to distinctly different size distributions (2 bimodal, 1 unimodal) and concentrations. The instruments showed agreement of ±2 nm for distribution mode and within ±10% for total number concentration.


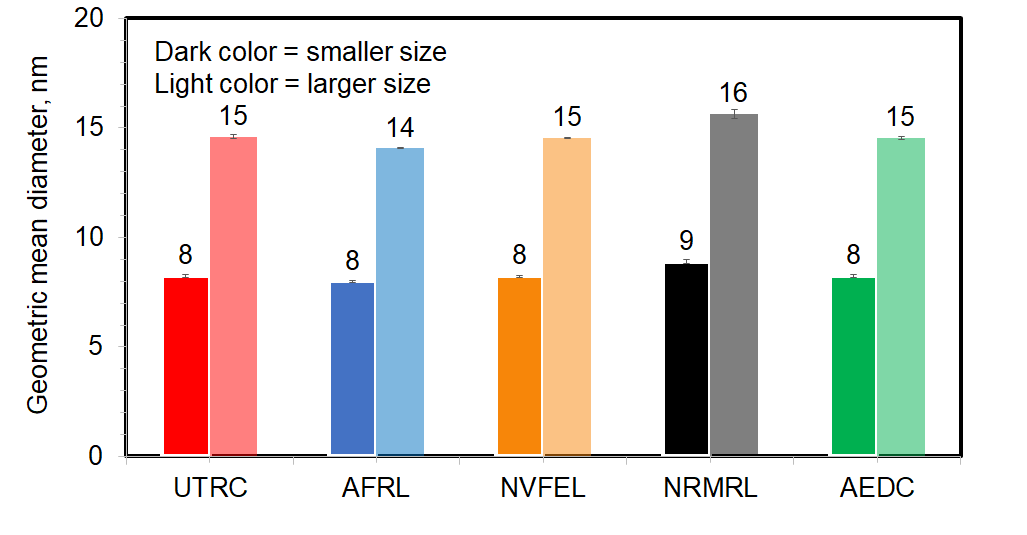

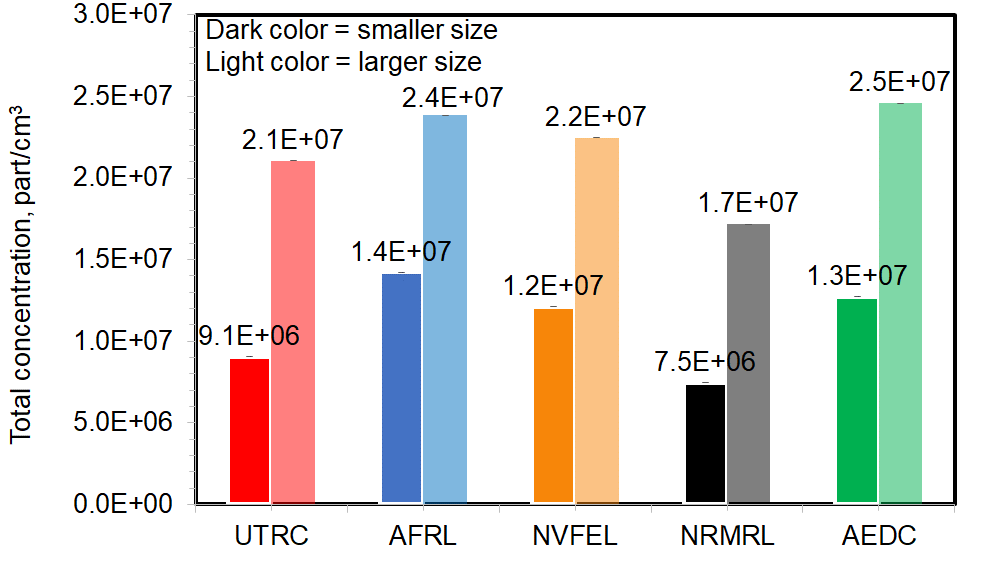


(b.)

(a.)

Figure S-6. Silver aerosol (a.) geometric mean diameter and (b.) total number concentration comparisons for 2 silver aerosol size distributions with geometric mean diameters of about 8 nm (smaller size/dark color), and 15 nm (larger size/light color).


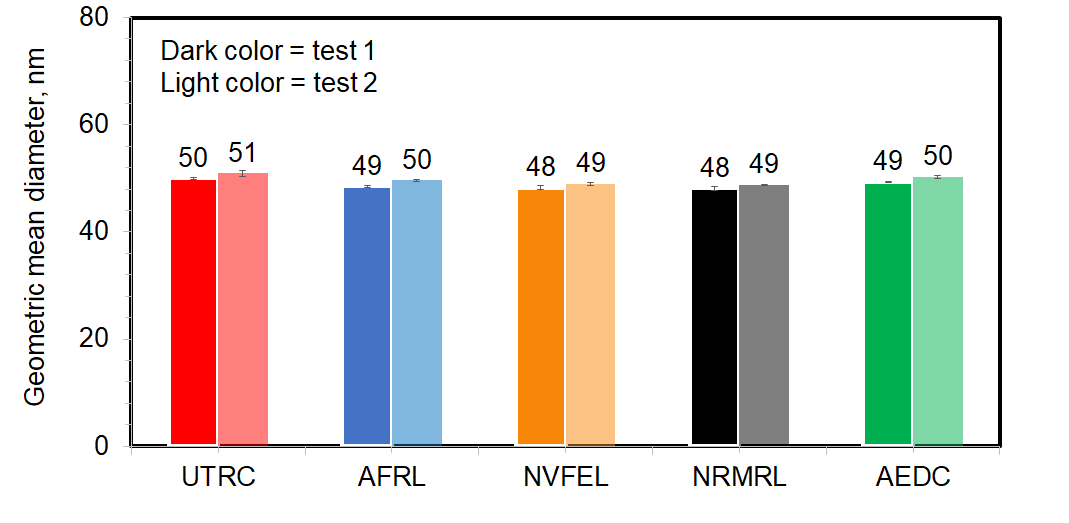

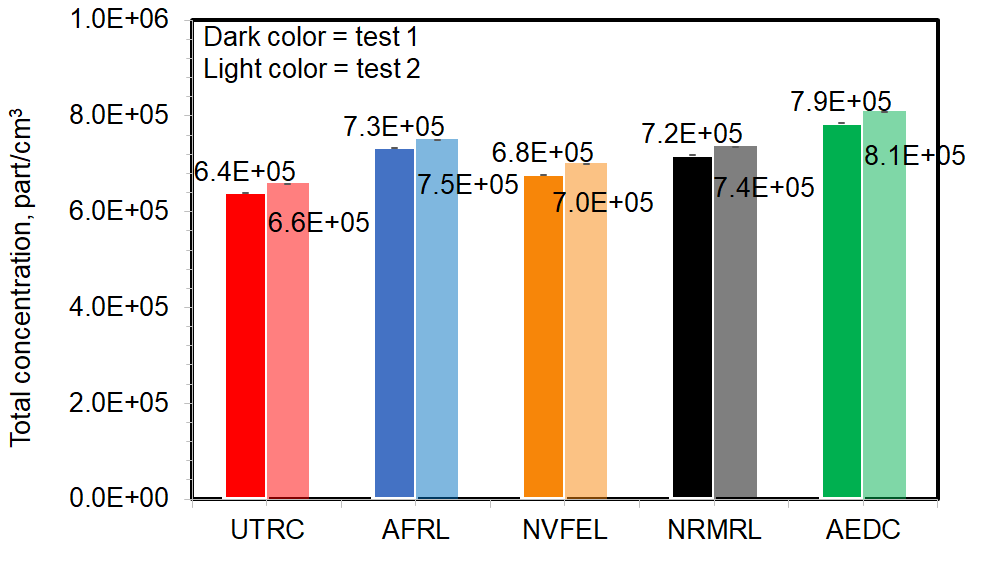


(b.)

(a.)

Figure S-7. DOS aerosol (a.) geometric mean diameter and (b.) total number concentration comparisons for DOS aerosol size distributions with geometric mean diameter of about 50 nm.


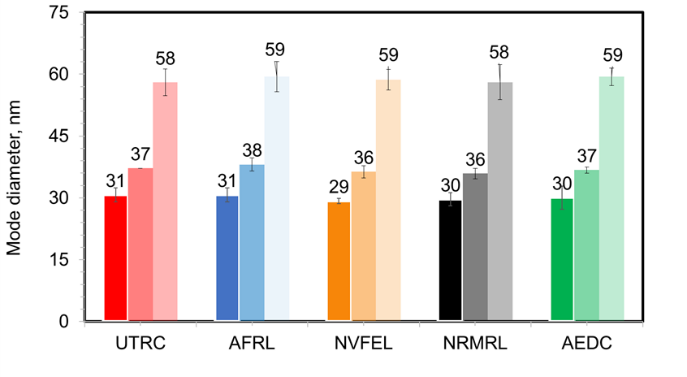

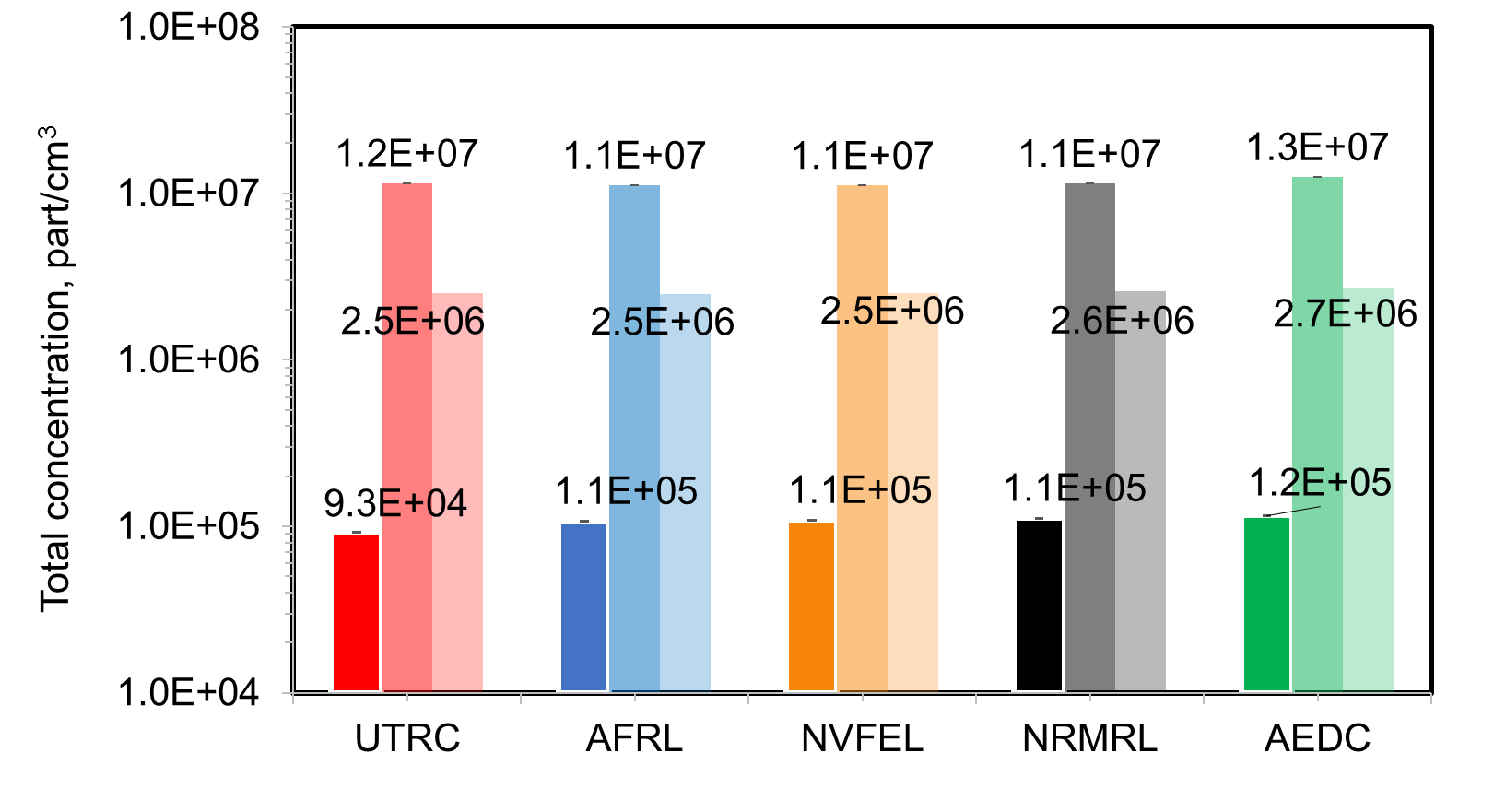


(b.)

(a.)

Figure S-8. Diesel soot (a.) mode diameter and (b.) total number concentration comparisons for 3 soot test conditions with mode diameters of about 30 nm, 37 nm, and 58 nm.

Finally, an aerosol of PSL in deionized water was generated in an atomizer produced as shown in Figure S-5. The NIST certified PSL spheres have mean diameter of 203±4 nm. To measure PSL spheres, the SMPS scan range was set from 170 to 230 nm to improve counting statistics. The measurements were made consecutively rather than concurrently. All 5 SMPSs agreed within 5% of expected PSL peak diameter.

### Onsite SMPS evaluations

After performing the pre-campaign measurements at the UMN, the instruments were sent to UTSI for the test campaign. Two additional instruments added to the test suite at UTSI: a TSI 3082 SMPS (loaner from TSI) and a TSI NanoScan^®^ (loaner from Jacob Swanson).

At UTSI another instrument response check was conducted prior to the beginning of testing, then during the tests the instruments were checked with a DOS aerosol. Finally, at the end of testing a comparison check of the instruments was done with a similar DOS aerosol. These tests and results are described below.


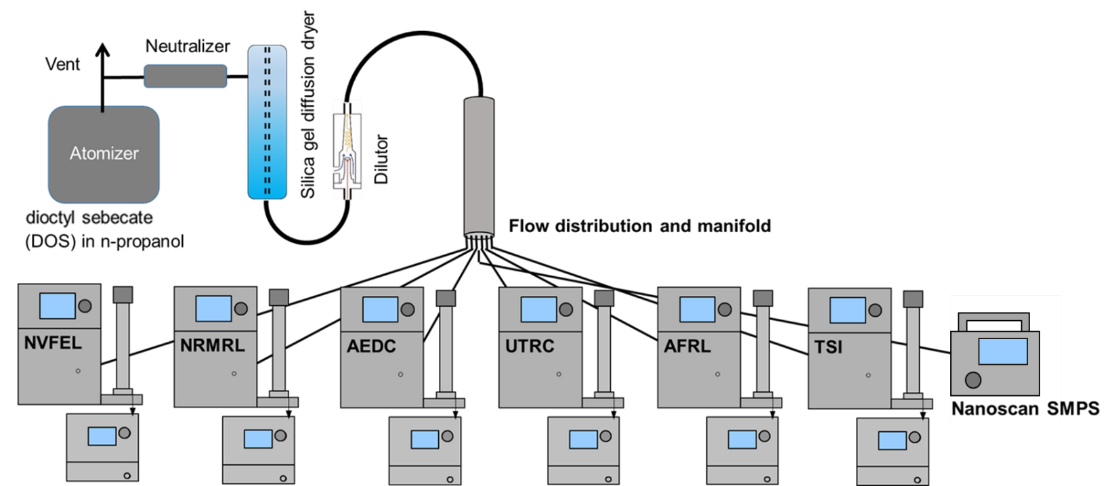


Figure S-9. Pre- and post- test DOS aerosol sampling configuration for UTSI onsite SMPS size distribution measurement comparisons. The NanoScan® was only used in the post test evaluation.


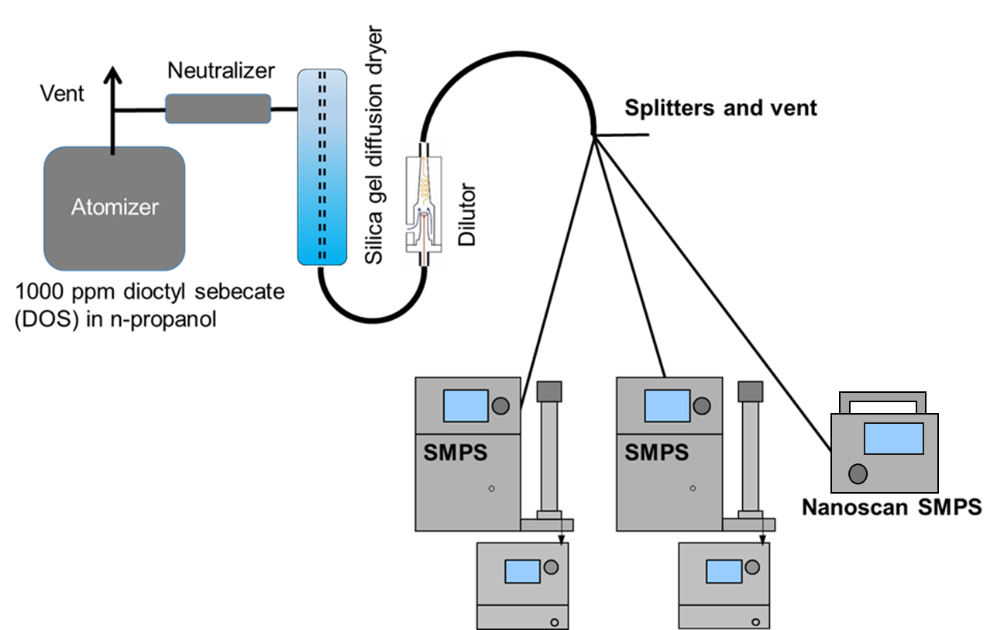


Figure S-10. Sampling configuration at UTSI for SMPS daily spot checks at UTSI. The NanoScan® was only used on 8/29 and 8/30.

Prior to the campaign, comparative size distribution measurements on DOS were performed for all instruments as shown in Figure S-9, which also included the TSI loaner instrument equipped with a 3082 classifier. The results of the on-site pre-campaign comparison are shown in Figure S-11 for particle size geometric mean diameter and total number concentration.


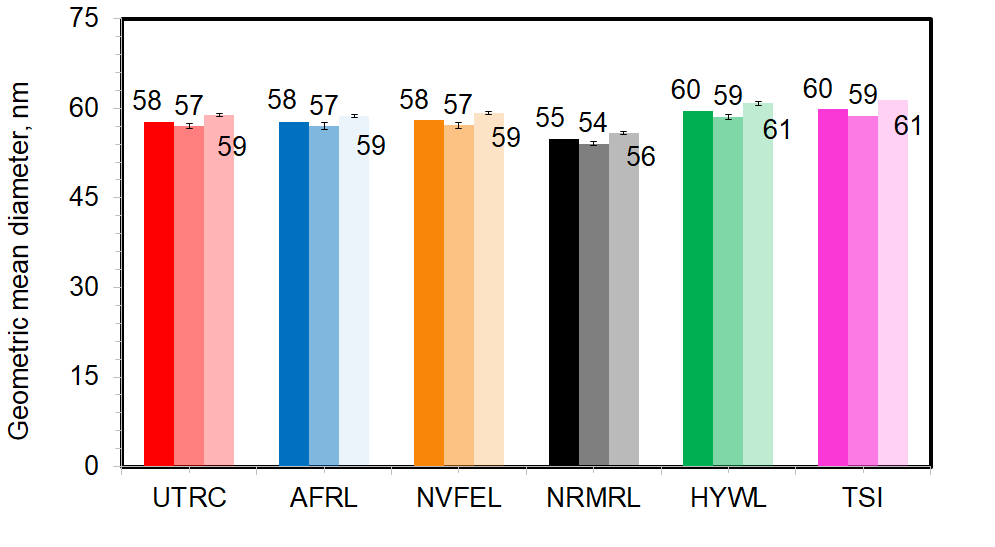

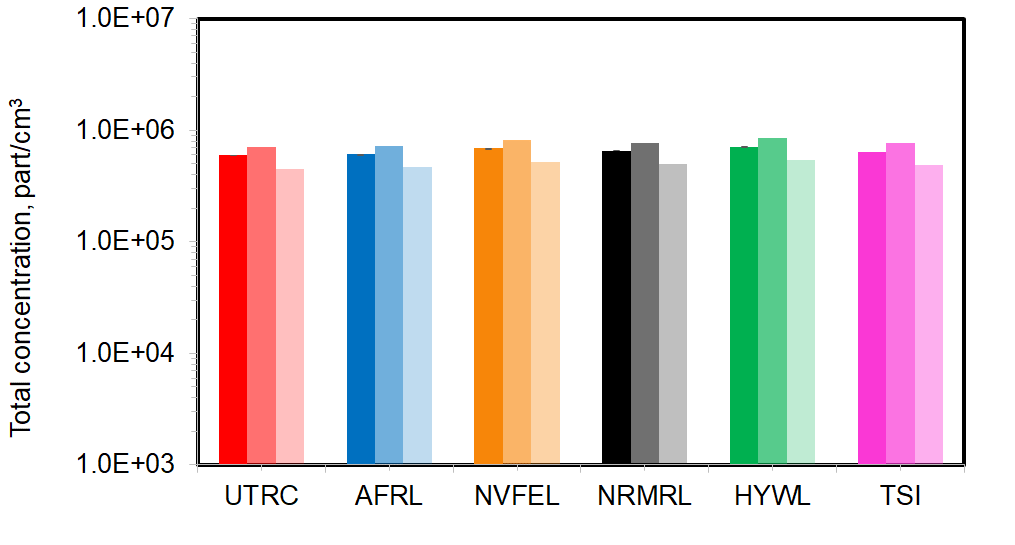


Figure S-11. DOS aerosol (a.) geometric mean diameter and (b.) total number concentration comparisons for 3 DOS aerosol size distributions. These comparisons were made onsite at UTSI.

The results of the all pre-campaign tests on SMPS confirmed that SMPS variability is within ±10% and that variability depends on particle size and composition. The SMPSs agreed better for larger particles. Additionally, very accurate CPC analog voltage output is critical to sizing performance at very small particle sizes and less important for larger sizes (e.g. >20 nm).

During the campaign, as time allowed, daily inter-comparisons of SMPS measured DOS aerosol size distributions were made. The DOS was aerosolized with a portable constant output atomizer. These tests compared the DOS aerosol size distributions measured by 2 SMPSs in a given sampling system along with the NanoScan^®^ SMPS, when available, as shown in Figure S-10. The NanoScan^®^ SMPS was portable, could be used in each sampling system, and could thus be used as a reference between all SMPSs. Results of these comparison are given in Table S-3.

Table S-3. DOS aerosol size distribution total number concentrations and geometric mean diameters for the SMPS comparison tests that were done on as many SMPSs as possible prior to the start of the engine tests.

| Date | Repeats | AEDC sampling system* | | | MS&T sampling system* | | | Near source sampling shed* | | |
| --- | --- | --- | --- | --- | --- | --- | --- | --- | --- | --- |
|  |  | AEDC | UTRC | NanoScan® | NRML | NVFEL | NanoScan® | AFRL | TSI | NanoScan® |
|  |  | Particle concentration (particles/cm^3^) / 10^5^  [geometric mean diameter (nm)] [geometric standard deviation] | | | | | | | | |
| 8/25/2015 | 1 | 4.51[39][1.8] | 4.62[39][1.8] | - | - | 5.70[39][1.8] | - | 10.5[51][1.7] | 11.1[50][1.7] | - |
|  | 2 | - | - | - | - | - | - | 4.87[37][1.8] | 5.24[36][1.8] | - |
| 8/26/2015 | 1 | 5.59[38][1.8] | 5.66[37][1.8] | - | - | 6.40[36][1.8] | - | 5.31[35][1.7] | 5.46[35][1.7] | - |
|  | 2 | - | - | - | - | - | - | 4.67[37][1.8] | 4.75[36][1.8] | - |
|  | 3 | - | - | - | - | - | - | 6.01[35][1.7] | 6.14[34][1.7] | - |
| 8/27/2015 | 1 | - | - | - | - | - | - | 5.28[39][1.7] | 5.36[39][1.7] | - |
| 8/29/2015 | 1 | 1.87[43][1.8] | 1.78[43][1.9] | 1.80[43][1.8] | - | 1.97[43][1.9] | 1.76[43][1.8] | 2.16[46][1.8] | 2.32[45][1.8] | 2.20[45][1.7] |
| 8/30/2015 | 1 | - | - | - | 2.96[49][1.8] | 2.60[50][1.8] | 3.00[50][1.7] | 2.58[49][1.8] | - | 2.89[47][1.7] |
|  | 2 | - | - | - |  |  |  | 2.66[50][1.8] | - | 2.97[49][1.7] |
|  | 3 | - | - | - |  |  |  | 2.73[51][1.8] | - | 3.03[49][1.7] |
| * Readings on the same row show SMPS comparisons simultaneously only for a single sampling system. | | | | | | | | | | |

On the final day of the test campaign, a comparison between all SMPSs including the NanoScan^®^ SMPS was performed as shown in Figure S-9. The instruments showed good day to day repeatability with concentrations within ~11% and geometric mean diameter within ~2 nm.

Several instruments malfunctioned during the test campaign. The AEDC TSI model 3776^®^ CPC was replaced with the TSI model 3776^®^ CPC from the NVFEL SMPS. The NVFEL SMPS then used a TSI model 3025a^®^ CPC for the remainder of the campaign. After switching CPCs, the NVFEL SMPS consistently read ~13% higher than other instruments. The NRMRL SMPS had issues measuring smaller particles during the campaign and was not used. The issue was determined to be the result of an older DMA which caused the smaller particles to be undermeasured.

# Thermophoretic losses

The thermophoretic loss calculation is a simplification of the formula found in (Yook and Pui 2005), (Tsai et al. 2004) and (Romay et al. 1998). It was developed in Kittelson and Johnson (1991) based on the formulation given by Hinds (1982; 1998) and Friedlander (1977; 2000). It assumes a well-mixed flow with a uniform concentration across the cross section of the tube except for a thin thermal boundary layer. For the sake of easy access, we reconstruct an argument for the use of the simplified form:

$\eta_{th}= \left( \frac{T_{w}}{T} \right)^{0.38}$ Equation S-1

Assumptions used by Kittelson and Johnson (1991) are as follows: well-mixed one-dimensional duct flow with no gradients in concentration or temperature normal to axial direction except in thin thermal boundary layer, negligible axial conduction, steady state, and steady flow. However, in cases where there are significant radial gradients across the cross section of the duct, such as in laminar flow, the concentrations near the thermal boundary layer may become depleted decreasing thermophoretic losses. In this case, the model here would overestimate the thermophoretic losses, but we believe based on experimental experience that this effect is negligible in most cases.

In Equations S-2 through S-6:

α= accommodation coefficient

*D_p_* = soot particle diameter

*C_p_* = specific heat

*dA*= wall area element

η_th_= penetration, the ratio of output to input concentration

*h* = carrier gas heat transfer coefficient

*k_g_* = carrier gas thermal conductivity

*K_n_* = 2λ/*D_p_* (Knudsen number)

*K_th_* = thermophoretic coefficient

λ = carrier gas mean free path

$\dot{m}$= mass flowrate

ν = kinematic viscosity

*Pr* = Prandtl number (= ρνC_p_/k_g_)

$\dot{Q}_{w}$= heat transfer rate to wall element *dA*

ρ= gas density

*r* = radial length

*T* = absolute temperature

*T_w_* = sample tube temperature

*V_d_* = thermophoretic deposition velocity

*x* = mass concentration

Mass transfer to wall by thermophoresis

$$\dot{m}x=\rho xV_{d}dA+\dot{m}(x+dx)$$

$\rho xV_{d}dA=-\dot{m}dx$ Equation S-2

For free molecular flow, Kn >>1 (Friedlander, 2000) gives

$$V_{d}=\frac{-3\nu\nabla T}{4\left( 1+\frac{\pi\alpha}{8} \right)T}$$

The only significant term in the thermal gradient, ∇T is the radial term, dT/dr, so that,

$V_{d}=\frac{-3}{4\left( 1+\frac{\pi\alpha}{8} \right)}\frac{\nu}{T}\frac{dT}{dr}=-K_{th}\frac{\nu}{T}\frac{dT}{dr}$ Equation S-3

where the thermophoretic coefficient (e.g., Waldmann and Schmitt, 1966),

$K_{th}\equiv\frac{3}{4\left( 1+\frac{\pi\alpha}{8} \right)} for K_{n}\gg1$

Then substituting Equation S-3 for V_d_ into Equation S-2

$K_{th}xdA\frac{\rho\nu}{T}\frac{dT}{dr}=\dot{m}dx$ Equation S-4

Convective heat transfer to wall

$$\dot{Q}_{w}=hdA\left( T-T_{w} \right)$$

together with the thermodynamic relation for heat transfer and C_p_

$$\dot{Q}_{w}=\frac{d}{dt}\left( C_{p}\rho VdT \right)=-\dot{m}C_{p}dT$$

yield

$hdA\left( T-T_{w} \right)=-\dot{m}C_{p}dT$ Equation S-5

From the Fourier law of heat conduction, the temperature gradient is proportional to the heat flux

$$\dot{Q}_{w}=-k_{g}\frac{dT}{dr}dA=hdA\left( T-T_{w} \right)$$

which gives the relation between the heat transfer coefficient, h, and thermal conductivity, k_g_,

$$h\left( T-T_{w} \right)=-k_{g}\frac{dT}{dr}$$

This can then be substituted into Equation S-5, yielding,

$$-k_{g}dA\frac{dT}{dr}=-\dot{m}C_{p}dT$$

Solving for, dT/dr,

$$\frac{dT}{dr}=\frac{\dot{m}C_{p}dT}{k_{g}dA}$$

Inserting the above expression for dT/dr into Equation S-4 and simplifying gives

$$K_{th}\frac{\rho\nu C_{p}}{k_{g}}\frac{dT}{T}=K_{th}\Pr\frac{dT}{T}=\frac{dx}{x}$$

then integrating gives

$$K_{th}Pr ln \left( \frac{T_{2}}{T_{1}} \right)=\ln\left( \frac{x_{2}}{x_{1}} \right)=\ln\left( \eta_{th} \right)$$

then exponentiating gives

$$\eta_{th}=\left( \frac{T_{2}}{T_{1}} \right)^{K_{th}Pr}$$

Using a = 0.9 (Friedlander, 2000) gives a value of K*_th_* = 0.554 and Pr for air = 0.69 (T < 700 K) give K*_th_*Pr = 0.382 so

$\eta_{th}=\left( \frac{T_{w}}{T} \right)^{0.382}$ Equation S-6

Note that the thermophoretic loss predicted by this model does not depend on the heat transfer coefficient or the wall temperature but only upon the inlet and outlet temperatures.

For larger particles, i.e., smaller Kn, K_th_ decreases. For example, at Kn = 1, D_p_ = 130 nm, K_th_ = 0.51 so the penetration becomes

$\eta_{th}=\left( \frac{T_{w}}{T} \right)^{0.352}$ Equation S-7

Taking an extreme case, a temperature decrease in the first section of the sampling system could be from 1000 to 433 K, the penetrations calculated by Equations S-6 and S-7 are 0.662 and 0.684, respectively, only about 3% difference.

The following will relate Equation S-1 and the above simplification to the thermophoretic penetration equation used in the UTRC model (Yook and Pui, 2005). The equation from the UTRC model is,

$\eta_{th}= \left( \frac{T_{w}+ \left( T- T_{w} \right)\cdot e^{- \frac{\pi D_{t}hL}{\rho QC_{p}}}}{T} \right)^{Pr\cdot K_{th}}={\left( \frac{T_{w}}{T} \right)^{Pr\cdot K_{th}}\cdot\left[ 1+ \left( \frac{T}{T_{w}}- 1 \right)\cdot e^{- \frac{\pi Nk_{g}L}{\rho QC_{p}}} \right]}^{Pr\cdot K_{th}}=\left( \frac{T_{w}}{T} \right)^{Pr\cdot K_{th}}\cdot f_{2}$ Equation S-8

with:

$f_{2}\equiv\left[ 1+ \left( \frac{T}{T_{w}}- 1 \right)\cdot e^{- \frac{\pi Nk_{g}L}{\rho QC_{p}}} \right]^{Pr\cdot K_{th}}$ Equation S-9

and where the thermophoretic coefficient, K_th_, has the form from Talbot, et al. (1980) that is applicable for all ranges of Kn (0 ≤ 2λ/D_p_ < ∞ ):

$K_{th}=\left( \frac{2\cdot C_{s}\cdot C_{C}}{1+3\cdot C_{m}\cdot K_{n}} \right)\cdot\left[ \frac{\left( \frac{k_{g}}{k_{p}} \right)+ C_{t}\cdot K_{n}}{1+2\cdot\left( \frac{k_{g}}{k_{p}} \right)+ C_{t}\cdot K_{n}} \right]$ Equation S-10

Also, in Equations S-1 and S-8 through S-10:

*C_m_* = 1.14 (soot momentum exchange coefficient from Yook and Pui 2005)

*C_p_* = sample carrier gas specific heat (in this work it is a parameterization in temperature

and pressure for air)

*C_s_* = 1.17 (thermal slip coefficient from Yook and Pui 2005)

*C_t_* = 2.18 (temperature jump coefficient from Yook and Pui 2005)

*D_p_* = soot particle diameter

*D_t_* = sample tube diameter

*K_n_* = 2λ/*D_p_* (Knudsen number)

*k_p_* = 0.2 J /m K (Yook and Pui 2005) (soot thermal conductivity)

*k_g_* = sample carrier gas thermal conductivity (in this work it is a parameterization in

temperature and pressure for air)

*K_th_* = thermophoretic coefficient [Tsai et al., 2004 (as with Friedlander, 2000) uses 0.55

for the free molecular region]

*L*=sample tube length

*Pr* = Prandtl number = C_p_ µ/ *k_g_*

*T* = inlet sample temperature in Kelvin

*T_w_* = sample tube temperature

*ρ* = gas density

*Re* = Reynolds Number

h = Ν *k_g_*/*D_t_* (convective heat transfer coefficient for fully developed turbulent flow

from Yook and Pui 2005)

Ν= *Ν*_0_ (*T/T_w_*)^0.36^  (from Yook and Pui 2005)

where:

${}_{0}= \left[ \frac{\left( \frac{\xi}{8} \right)\cdot\left( Re-1000 \right)Pr}{1 + 1.27\cdot\left( {Pr}^{2/3}-1 \right)\cdot\sqrt{\frac{\xi}{8}}} \right]\cdot\left[ 1+\left( \frac{D_{t}}{L} \right)^{2/3} \right]$ (from Yook and Pui 2005)

$\xi=\frac{1}{\left[ 1.82\cdot{log}_{10}\left( Re \right)-1.64 \right]^{2}}$ (from Yook and Pui 2005)

$\lambda= \lambda_{0}\cdot\left( \frac{T}{T_{0}} \right)\cdot\left( \frac{P_{0}}{P} \right)\cdot\left[ \frac{1+\left( \frac{110.4}{T_{0}} \right)}{1+\left( \frac{110.4}{T} \right)} \right] , \lambda_{0}=67.3 nm, T_{0}=296.15 K, P_{0}=101325 Pa$ (carrier gas mean free path, λ, from Willeke 1976)

$\mu= \mu_{0}\cdot\left( \frac{T}{T_{0}} \right)^{3/2}\cdot\left( \frac{T_{0}+110.4}{T+110.4} \right) , \mu_{0}=1.83245 \times{10}^{-5}kg/(m\cdot s), T_{0}=296.15 K, and P_{0}=101325 Pa$ (carrier gas viscosity, $\mu$, from Willeke 1976)

The expression for f_2_, Equation S-9, is plotted as a function of inlet gas temperature, T, and as a function of the ratio of the inlet gas temperature and the sample tube wall temperature, T/T_w_, in Figures S-12a and Figure S-12b, respectively for three different sample flows, 30 liters/minute, 50 liters/minute, and 100 liters/minute. For these gas flows and for temperatures below 500 K (gas temperature ratios, T/T_w_, from 1 to 1.8), the function f_2_ changes from 1 to 1.05 (a 5% difference) and is nearly independent of the gas flow. Aircraft engine exit plane exhaust temperatures can reach near 1200 K. The sample line temperature from the exhaust plane to the diluter is held at 433.15 K (see the sampling system diagram in Figure 1). The applicable sample gas temperature to sample line wall temperature ratios will be in the range, T/T_w_ < 2.3.


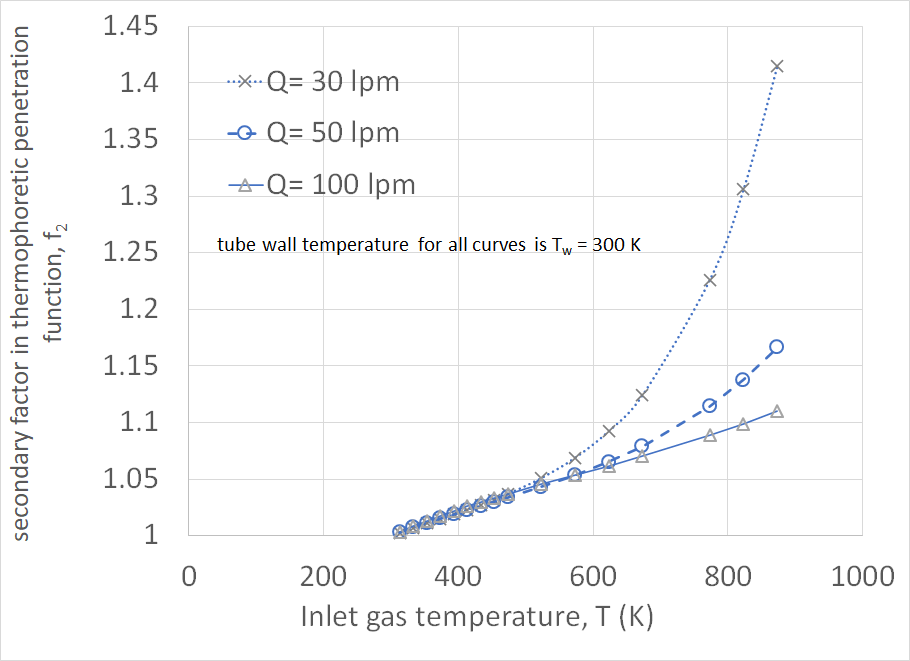

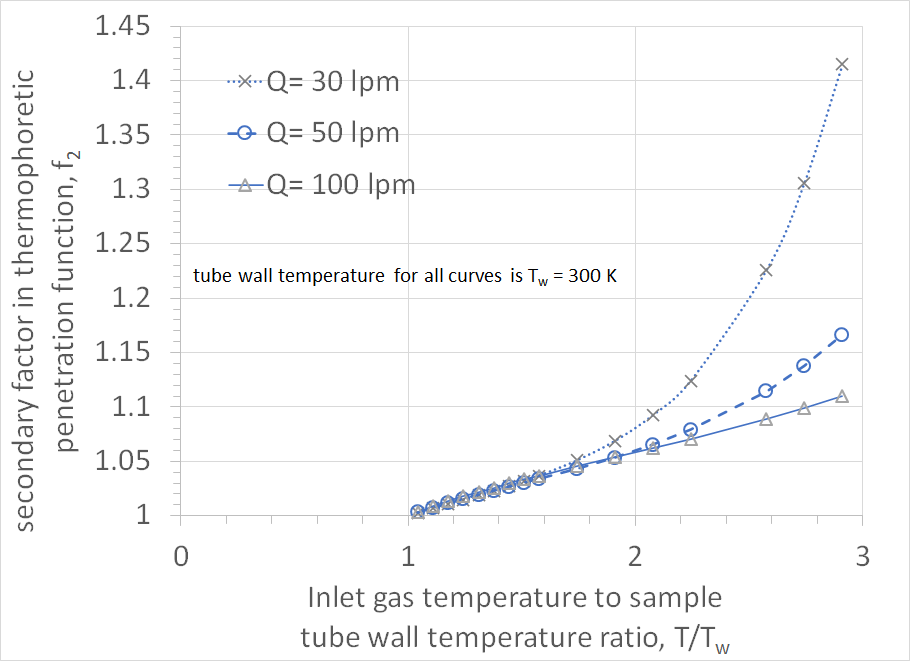


Figure S-12. The function, f_2_, (Equation S-9) as a function of (a) inlet sample carrier gas temperature and (b) the ratio, T/T_w_, of the inlet sample carrier gas temperature and the sample tube wall temperature. The function deviates from 1 as the ratio, T/T_w_, increases above 1 linearly for all flow rates 30 lpm and greater until approximately T/T_w_ =1.8 where for Q=30 lpm the increase has quadratic or higher order dependence on T/T_w_. At this point the function f_2_≈1.05 or has about a 5% difference from 1.


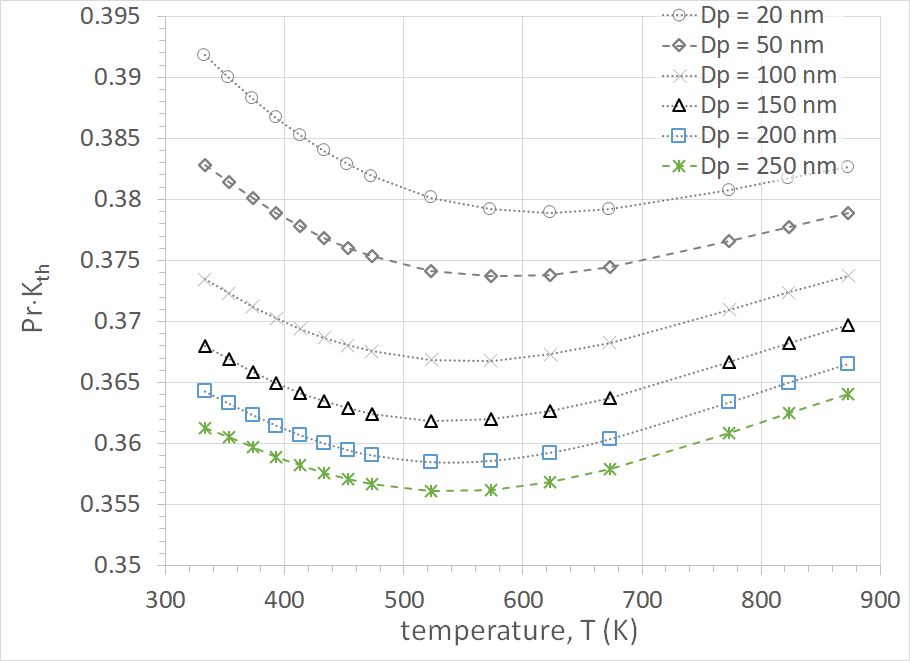


Figure S-13. Temperature and particle diameter dependencies of the exponential factor, Pr⋅K_th_, in Equation S-8. The particle diameter dependence is the more important of the dependencies and the value of 0.38 (see text) used in the simplified form is applicable for particles with diameters **≲** 100nm.

In Figure S-13 the product of the Prandtl number, Pr, and the thermophoretic coefficient, K_th_ (=0.55 from Tsai et al. 2004), is graphed as a function of carrier gas temperature for particle diameters ranging from 20 nm to 250 nm. For particles with diameters 20 nm to 100 nm the product varies from a maximum of about 0.393 to a minimum of about 0.367, a relative difference of about 7%. From Hinds, the Prandtl number for particles in the free-molecular range (diameters less than 100 nm) is approximately, 0.7. Hence the approximate value of the product, K_th_⋅Pr = 0.38, as first used by Kittelson and Johnson (1991).

Considering the above, i.e., f_2_ ≈ 1 and Pr⋅K_th_ ≈ 0.38, the Tsai et al. (2004), formulation can be simplified with Equation S-1, as first proposed by Kittelson and Johnson (1991), for particles with diameters <10nm and respecting assumptions mentioned above about flows, gas and tube temperatures. Figure S-14 illustrates the difference between the exact thermophoretic expression from Tsai et al. in Equation S-8, and the simplified, approximate expression of Equation S-1. The graph illustrates the particle diameter and temperature ranges for which this approximation is applicable.


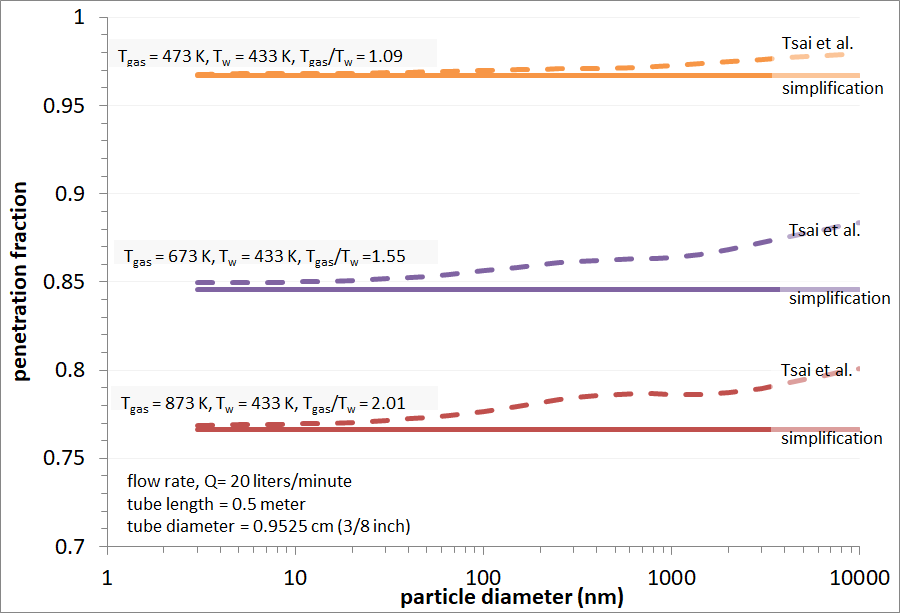

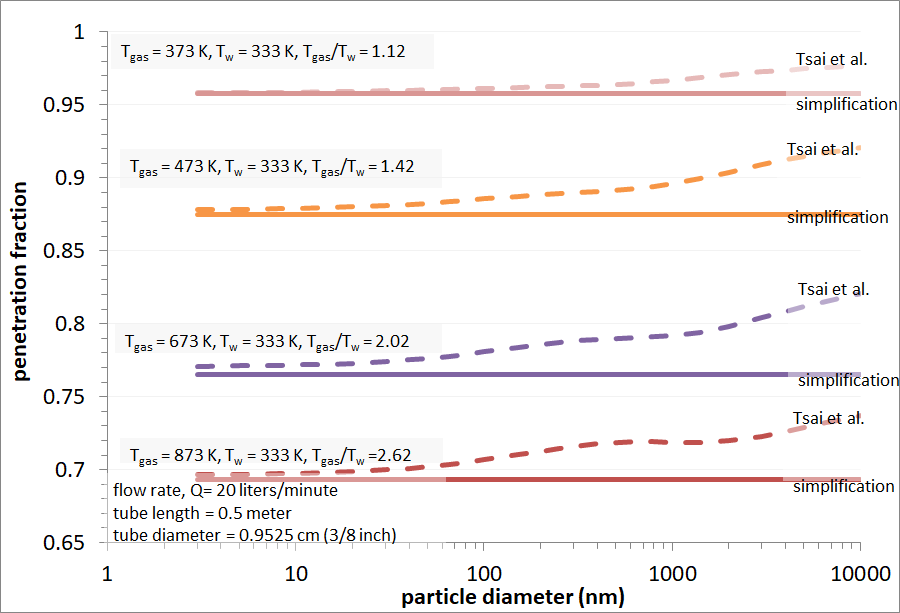


Figure S-14. As indicated in Figure S-13, the discrepancy between the simplified thermophoretic penetration (Equation S-1) begins for particles with diameters **≳** 100nm. Additionally, the deviations of the simplified formula from the Tsai et al., 2004, formula (Equation S-8) due to the increasing temperature ratio, T/T_w_, can also be seen.

# Normalization of instrument responses and line length differences

Considerations for different responses in SMPSs and differences in connecting line losses were made to determine the sampling system component particle penetration more accurately. A normalization of the downstream SMPS to the upstream SMPS instrument response was determined. Additionally, care was taken to minimize and account for losses in all connecting lines.

## Normalization of SMPS instrument responses

The relative instrument response between the upstream and downstream SMPS was determined by measuring DOS aerosol size distributions generated from an atomizer. The DOS aerosol was supplied to each SMPS via a plenum and sample lines from the plenum to each SMPS were of equal length, diameter, and material (carbon-impregnated Teflon) as illustrated in Figure S-9. Additionally, the AEDC and UTRC SMPSs were compared during the campaign as shown in Figure S-10 as part of the daily checks.

The normalization factor for the SMPSs used in the evaluation of the 25-m line was the ratio of the average of 22 size distribution scans from the upstream AFRL SMPS and downstream AEDC SMPS as shown in Figure S-15. The normalization factor for the SMPSs used in the evaluation of the VPR was the ratio of the average of 33 size distribution scans from the upstream AEDC SMPS and downstream UTRC SMPS as shown in Figure S-16. The ratio was then fit to an exponential function, e.g., solid light blue line in Figure S-15(b) and Figure S-16 (b), and the fit values at different particle diameters were applied to normalize the downstream SMPS responses to the upstream SMPS. The fit showed that the response between the SMPSs was better than the 5% for particle diameters larger than 10 nm.


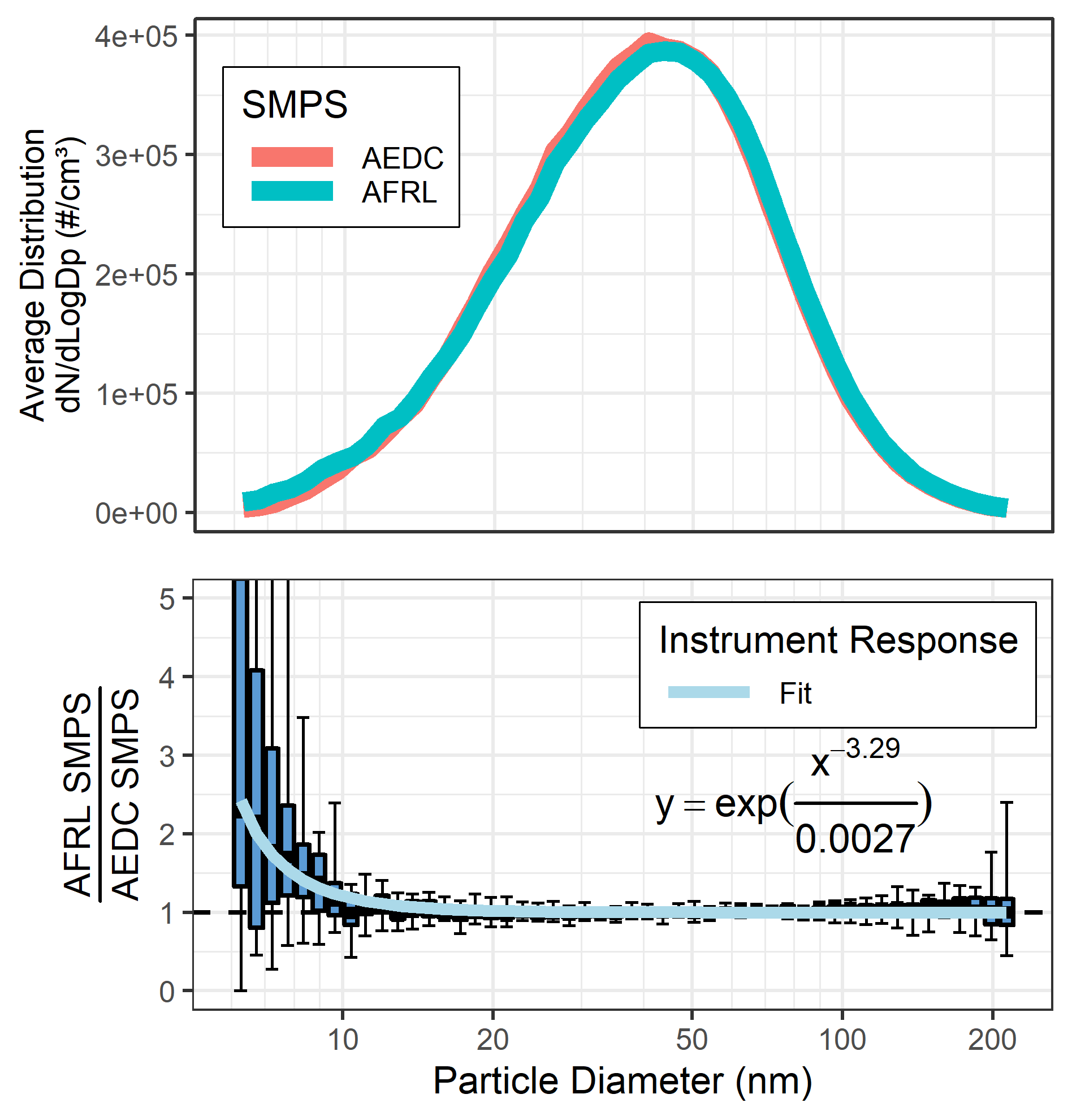


Figure S-15. (a) AEDC SMPS(red line), and AFRL SMPS(solid light blue line) average size distribution for 22 scans of DOS aerosol size distributions used to determine the (b) normalization ratio, N_AFRL/AEDC_(D_p_), determined by taking the ratio of the AFRL to the AEDC SMPS measured size distributions from (a). In (b), the solid light blue line is an exponential fit to the individual ratios at each SMPS diameter.


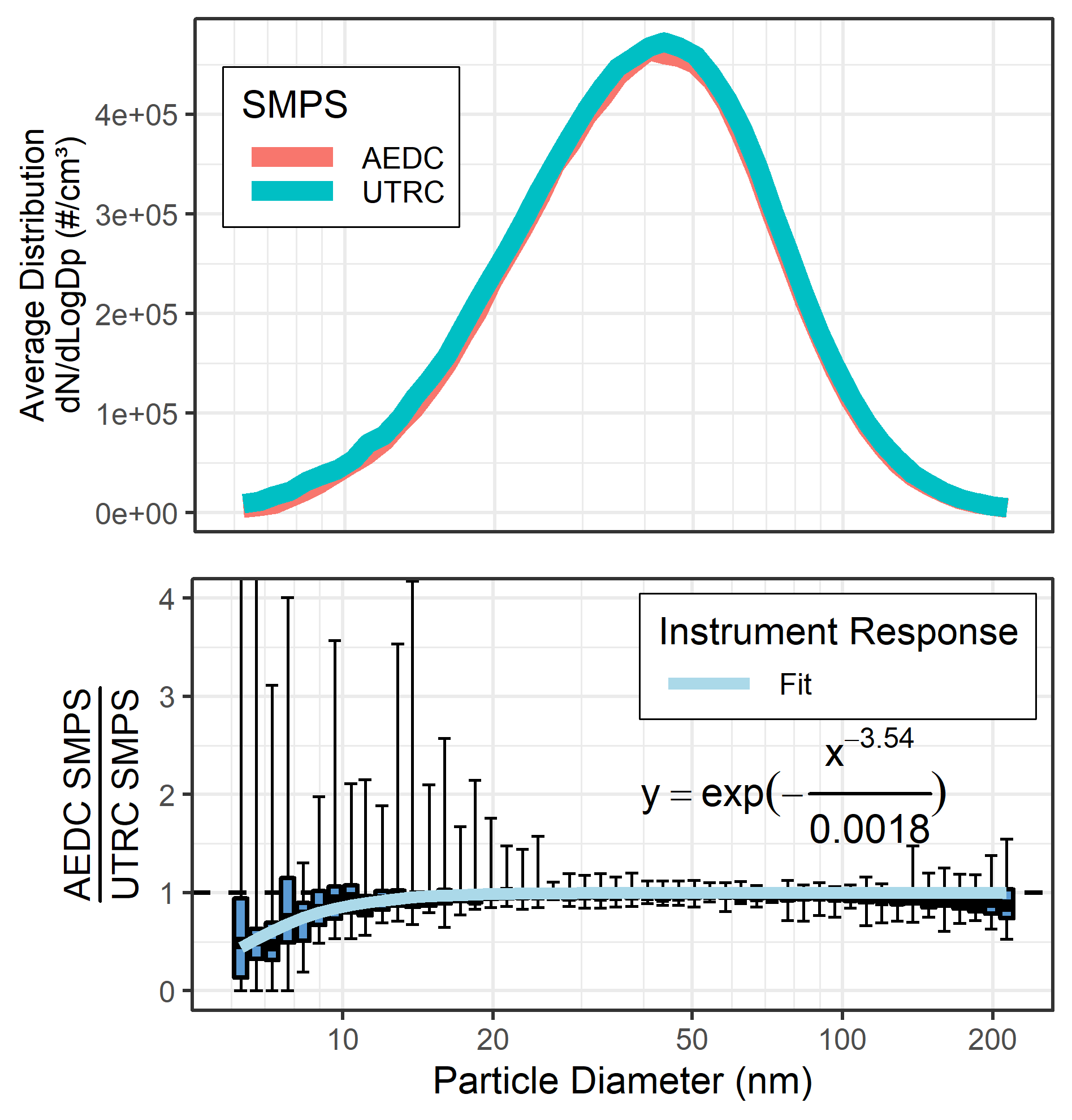


Figure S-16. (a) AEDC SMPS(red line) and UTRC SMPS (solid light blue line) average size distribution for 33 scans of DOS aerosol size distributions used to determine the (b) size dependent normalization ratio, N_AEDC/UTRC_(D_p_) determined by taking the ratio of the AEDC to the UTRC SMPS measured size distributions from (a). In (b), the solid light blue line is an exponential fit to the individual ratios at each SMPS diameter.

## Sampling system component inlet and outlet to SMPS sample line differences

The connecting line lengths from the inlet and outlet of the 25-meter line to the AFRL and AEDC SMPSs, respectively, had slightly different lines. Penetrations for each of these lines were calculated using the UTRC penetration calculation tool. The penetrations calculated (solid blue and yellow lines) are illustrated in Figure S-17 along with the normalization factor (pink dashed line) determined by taking the ratio of the upstream AFRL SMPS sample line penetration to the downstream AEDC SMPS sample line penetration.


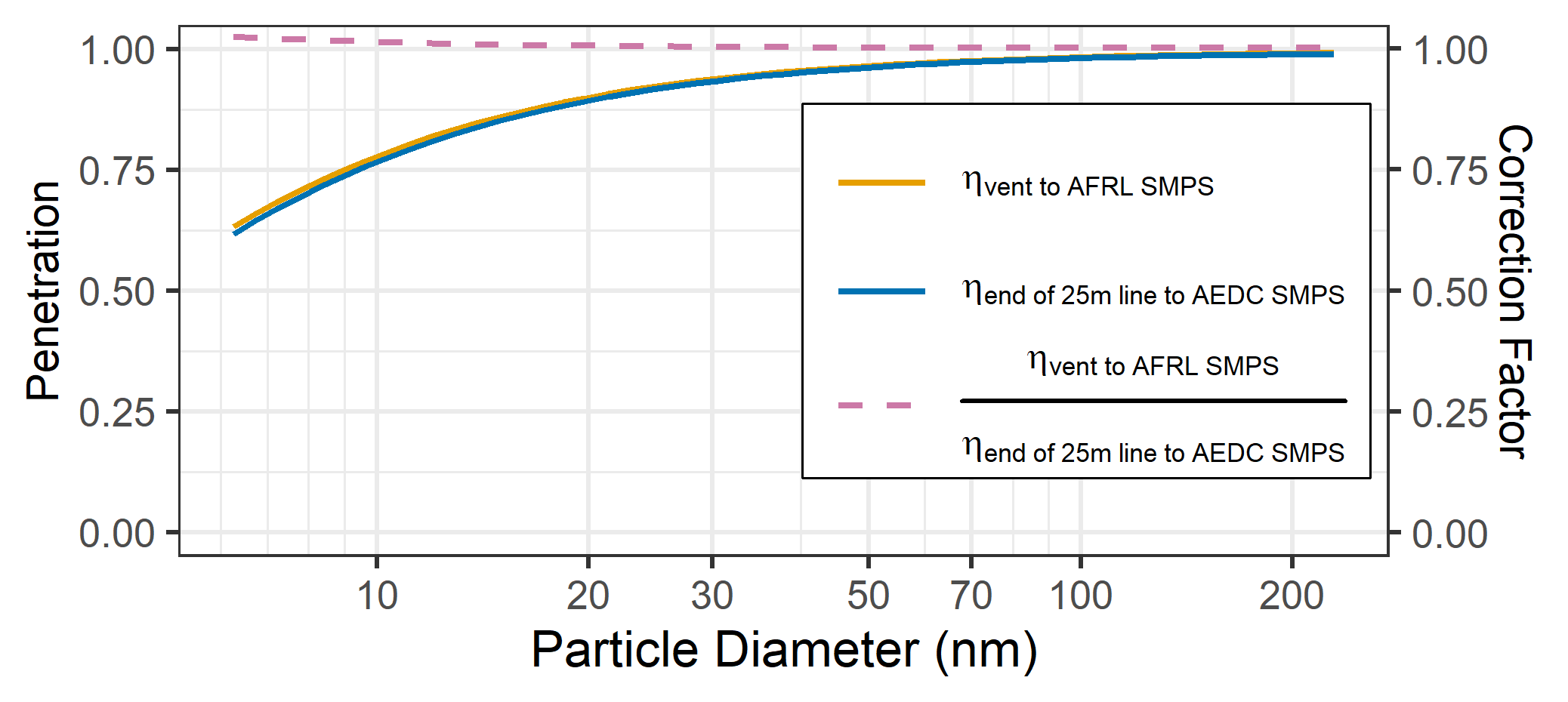


Figure S-17. Sample lines from the inlet and outlet of the sampling system components to the SMPSs were not identical as shown in Figure 2b of the main text. To account for any differences in the size distributions measured by the SMPSs due to these sample lines, calculations of penetration efficiencies in these additional sample lines were made and then compared to one another. The ratio (dashed line) was used to as a correction factor to the measured penetration of the 25-m line.


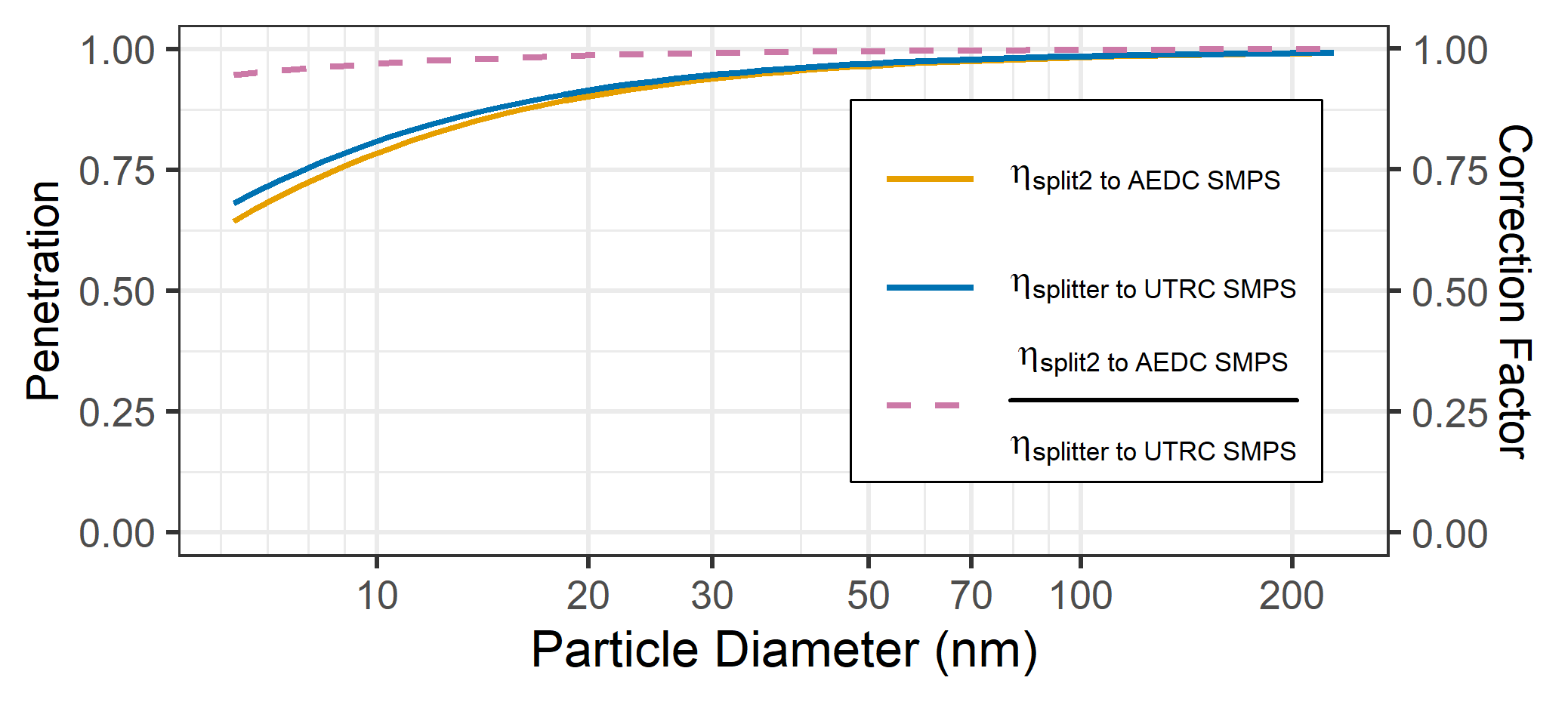


Figure S-18. Sample lines from the inlet and outlet of the sampling system components to the SMPSs were not identical as shown in Figure 2b of the main text. To account for any differences in the size distributions measured by the SMPSs due to these sample lines, calculations of penetration efficiencies in these additional sample lines (solid lines) were made and then compared to one another. The ratio (dashed line) was used to as a correction factor to the measured penetration of the VPR.

The connecting line lengths from the inlet and outlet of the VPR to the AEDC and UTRC SMPSs, respectively, had slightly different lines. Penetrations for each of these lines were calculated using the UTRC penetration calculation tool. The penetrations calculated (solid blue and yellow lines) are illustrated in Figure S-18 along with the normalization factor (pink dashed line) determined by taking the ratio of the upstream AEDC SMPS sample line penetration to the downstream UTRC SMPS sample line penetration.

# 25-meter line configuration

For purposes of assessing penetration of the 25-m sampling line, the physical configuration was characterized. Table S-4 shows the line lengths, bends, etc. of the sampling line characterized in this study.

Table S-4: Lengths, bends, inlets, and outlets of the heated 25 meter carbon-impregnated Teflon line with a diameter of 0.8 cm in the AEDC sampling system held at a temperature of 60 °C.

| **Length (cm)** | **Bend Angle (degrees)** | **Bend Radius (cm)** |
| --- | --- | --- |
| 15 | 45 | 20 |
| 38 | straight | – |
| 41 | 45 | 102 |
| 64 | straight | – |
| 51 | 20 | 127 |
| 140 | straight | – |
| 89 | 30 | 152 |
| 51 | straight | – |
| 244 | 360 | 36 |
| 244 | 360 | 36 |
| 213 | 45 | 81 |
| 236 | straight | – |
| 127 | 45 | 127 |
| 36 | 45 | 51 |
| 224 | straight | – |
| 25 | 90 | 15 |
| 33 | straight | – |
| 114 | 90 | 51 |
| 25 | 90 | 13 |
| 229 | straight | – |
| 56 | 90 | 25 |
| 56 | 45 | 51 |
| 61 | straight | – |
| 36 | 45 | 51 |
| 25 | 45 | 25 |
| 23 | straight | – |

# Normalized measured median penetration compared to modeled penetration for 25 m line and VPR

Figure S-19 shows a comparison between the median of the measured penetrations and the 25-m line modeled penetrations for the test conditions shown in Figure 3 of the main text. The ratios in Figure S-19 show quantitatively how well the measured penetrations agreed with the modeled penetrations and that between 7 and 120 nm, the penetrations measured for the 25-m line agree with values predicted by the UTRC line loss model to within ±15%.


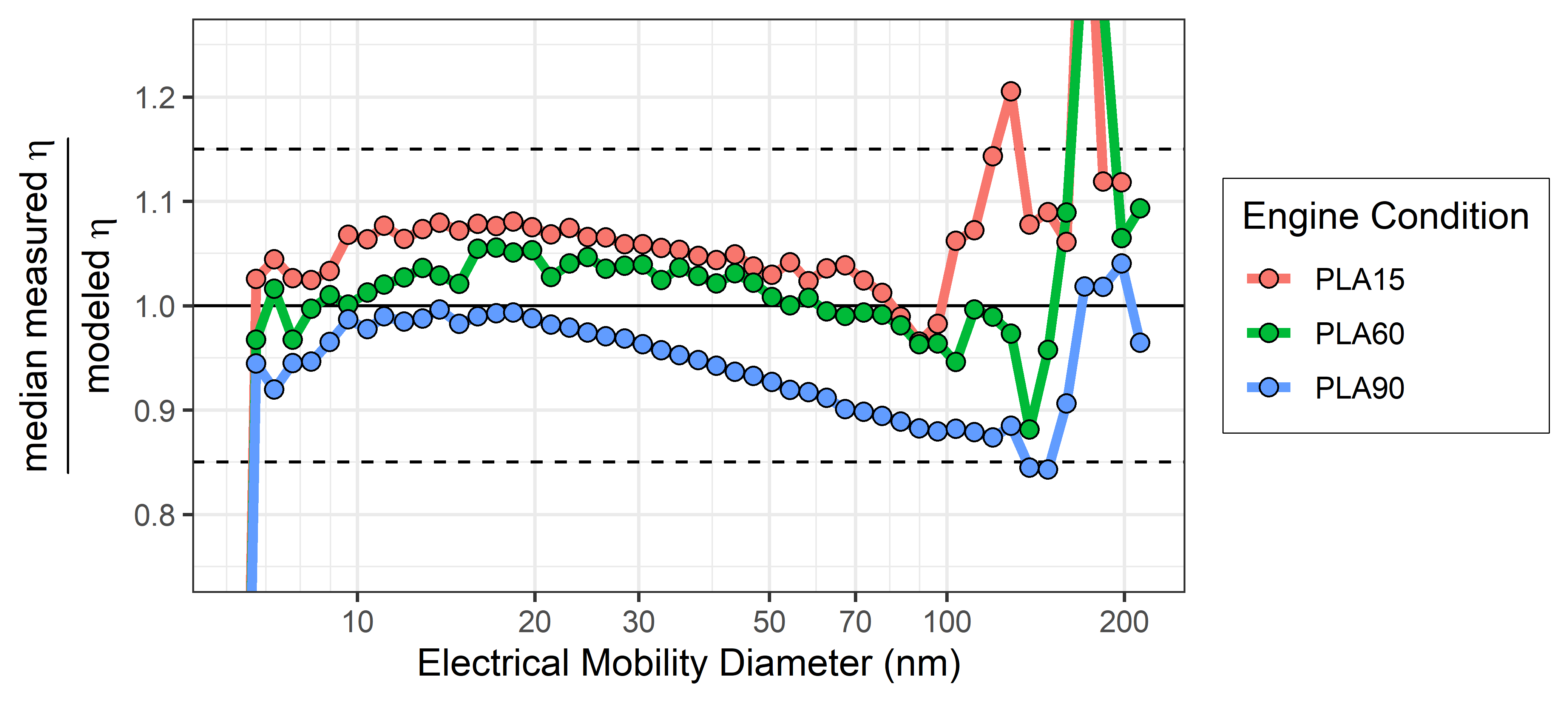


Figure S-19. Ratio of the measured median penetrations to the modeled penetrations for the 3 test conditions in Figure 3 of the main text. The black solid horizontal line shows a 1:1 agreement and the dashed horizontal lines show the bounds of ±15% from 1:1 agreement.

Figure S-20 shows a comparison between the measured median penetration and the modeled penetration for the VPR for the test condition shown in Figure 4 of the main text. The ratios in Figure S-20 shows quantitatively how well the measured penetrations agreed with the modeled penetrations. There is scatter due to the low concentrations measured by the SMPS after the VPR, but where the errors are reasonable, i.e. between 11 and 50 nm, the data and the model agree to within 50%.


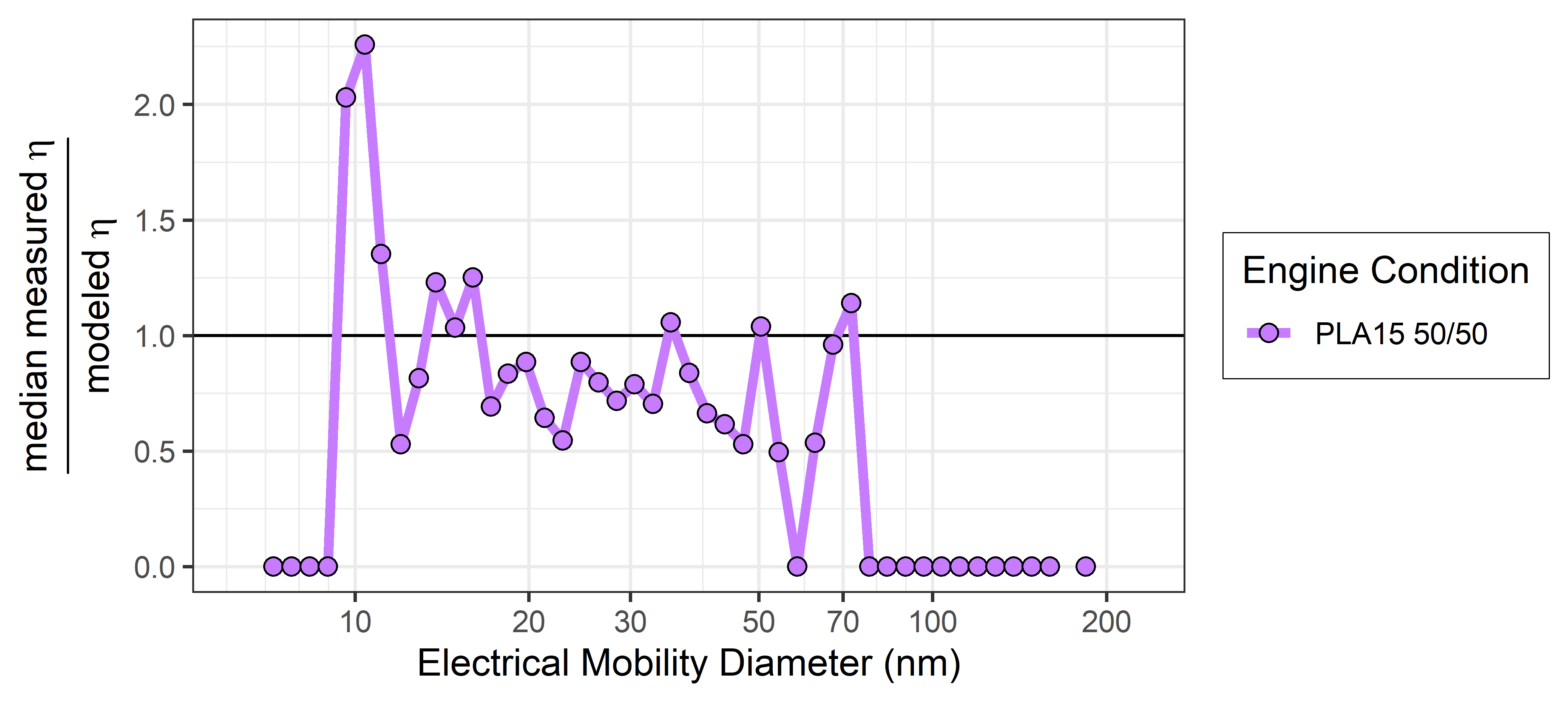


Figure S-20. Ratio of the median measured penetrations to the modeled penetrations for the test condition in Figure 4 of the main text. The black solid horizontal line shows a 1:1 agreement.

7. References

Friedlander, Sheldon K. 1977. *Smoke, Dust, and Haze*. 1st ed. New York: Wiley-Interscience.

Friedlander, Sheldon K. 2000. *Smoke, Dust, and Haze*. 2nd ed. New York: Oxford University Press.

Hinds, W. C. 1982. *Aerosol Technology: Properties, Behavior, and Measurement of Airborne Particles*. 1st ed. New York: John Wiley & Sons.

Hinds, W. C. 1999. *Aerosol Technology: Properties, Behavior, and Measurement of Airborne Particles*. 2nd ed. New York: John Wiley & Sons.

Kittelson, D. B., and J. H. Johnson 1991. Variability in Particle Emission Measurements in the Heavy Duty Transient Test, SAE Paper Number 910738.

Romay, F. J., S. S. Takagaki, D. Y. H. Pui, and B. Y. H. Liu. 1998. Thermophoretic Deposition of Aerosol Particles in Turbulent Pipe Flow. *Journal of Aerosol Science* 29 (8): 943–59. <https://doi.org/10.1016/S0021-8502(98)00004-4>.

Talbot, L., R. K. Cheng, R. W. Schefer, and D. R. Willis. 1980. Thermophoresis of particles in a heated boundary layer, *J. Fluid Mech.*, 101, part 4, pp. 737-758

Tsai, C.-J., J.-S. Lin, S. G. Aggarwal, and D.-R. Chen. 2004. Thermophoretic Deposition of Particles in Laminar and Turbulent Tube Flows. *Aerosol Science and Technology* 38 (2): 131–39.

Waldmann, L. and K.H. Schmitt. 1966. Aerosol Science (Edited by Davies, C. N.), Chapter 6. Academic Press, New York.

Willeke, K. 1976. Temperature Dependence of Particle Slip in a Gaseous Medium. *Journal of Aerosol Science* 7 (5): 381–87. <https://doi.org/10.1016/0021-8502(76)90024-0>.

Yook, S. J., and D. Y. H. Pui. 2005. Estimation of Penetration Efficiencies through NASA Sampling Lines. *Submitted to C.-M. Lee, NASA Glenn Research Center*.
